# Health care utilization before and after COVID-19 diagnosis: a multidisease matched-cohort study by sociodemographic factors

**DOI:** 10.1186/s12913-026-14934-z

**Published:** 2026-06-24

**Authors:** Alina Peluso, Omar Aljawfi, Kelley M. Anderson, Margret V. Bjarnadottir, Nawar Shara

**Affiliations:** 1 Oak Ridge National Laboratory, Oak Ridge, TN 37830 USA; 2 MedStar Health Research Institute, Hyattsville, MD 21044 USA; 3 University of Virginia, Charlottesville, VA 22903 USA; 4 University of Maryland, College Park, MD 20742 USA

**Keywords:** COVID-19, Pandemic, Electronic Health Records (EHR), Healthcare utilization, Chronic conditions, Mental health, Demographics, Socioeconomic factors, Excess visits, Difference-in-differences (DiD), Telehealth

## Abstract

**Background:**

The COVID-19 pandemic substantially disrupted healthcare utilization across clinical conditions, populations, and care settings. Consistent and equitable access to care is essential to mitigate the long-term consequences of COVID-19, particularly among individuals with pre-existing conditions. While prior studies have documented broad patterns in healthcare use during the pandemic, limited evidence exists on how utilization shifts before and after a COVID-19 diagnosis, particularly across specific diseases, encounter types, and sociodemographic subgroups.

**Methods:**

We conducted a matched retrospective cohort study using electronic health record data from MedStar Health between March 25, 2019, and June 30, 2021. A difference-in-differences design was used to compare changes in health care utilization between adult patients who tested positive for COVID-19 (exposure group) and those who tested negative (comparison group), across the 6-month periods before and after testing. Outcomes included encounters across outpatient, inpatient, emergency department, and virtual settings, overall and stratified by pre-existing conditions (heart failure, diabetes mellitus, kidney disease, malignant neoplasm, hypothyroidism, hyperthyroidism, anxiety disorder, depressive disorder, and drug poisoning/overdose), while accounting for demographic characteristics (sex, age, race/ethnicity, insurance type, state of residence) and census tract–level socioeconomic indicators (median household income, educational attainment, and poverty/food access). To estimate the absolute burden of health care utilization associated with COVID-19, we converted DiD rate ratios into excess encounters per 1,000 individuals in the exposed group.

**Results:**

COVID-19 exposure was associated with a 4% overall reduction in health care utilization during the post-COVID period (RR = 0.96, *p* < 0.001, 262 fewer visits per 1,000 patients), primarily driven by decreased outpatient visits (RR = 0.96, *p* < 0.001). Significant declines were observed among adults aged ≥ 65 years (RR = 0.92, *p* < 0.001), males (RR = 0.90, *p* < 0.001), Black individuals (RR = 0.96, *p* < 0.001), and Medicare beneficiaries (RR = 0.91, *p* < 0.001). Geographically, encounter rates declined significantly in DC (RR = 0.93, *p* < 0.001) and Maryland (RR = 0.98, *p* = 0.023), but not in Virginia. Socioeconomic indicators also correlated with healthcare utilization: patients residing in areas with lower income (RR = 0.94, *p* = 0.003), lower educational attainment (RR = 0.95, *p* = 0.011), or higher rates of food stamp/SNAP participation (RR = 0.93, *p* < 0.001) experienced greater declines in utilization. Among clinical conditions, the largest reductions in healthcare visits were seen for patients with kidney disease (RR = 0.88, *p* < 0.001), drug poisoning/overdose (RR = 0.88, *p* < 0.001), hypothyroidism (RR = 0.91, *p* < 0.001), diabetes mellitus (RR = 0.94, *p* < 0.001), and heart failure (RR = 0.94, *p* = 0.002). In contrast, encounters for patient cohorts with hyperthyroidism (RR = 1.14, *p* = 0.004) increased notably, while those for depressive disorders (RR = 1.03, *p* = 0.072) showed a modest increase. No significant changes were observed in patient cohorts with anxiety (RR = 1.00, *p* = 0.83) and cancer (RR = 1.00, *p* = 0.81).

**Conclusions:**

COVID-19 exposure was associated with broad but varied changes in healthcare utilization during the post-COVID period. Several chronic conditions showed notable declines in encounter rates, although these patterns were not consistent across all conditions. The heterogeneity in utilization patterns suggests potential gaps in ongoing care and highlights the need for targeted efforts to support continued management of health conditions after COVID-19.

**Supplementary Information:**

The online version contains supplementary material available at 10.1186/s12913-026-14934-z.

## Key points

**Question**

How does healthcare utilization before and after a COVID-19 diagnosis vary by disease cohorts, encounter type, and sociodemographic characteristics?

In this multidisease matched-cohort study, COVID-19 diagnosis was associated with reduced healthcare use, particularly in outpatient settings. Declines were most pronounced among older adults, male, Black individuals and Medicare beneficiaries, and varied with patients’ underlying conditions, with kidney disease and diabetes patients’ utilization declining the most.

These findings highlight variation in healthcare access and use surrounding COVID-19 diagnosis, emphasizing the need for targeted interventions to support effective, disease-specific recovery and chronic disease management in the post-acute phase of COVID-19.

## Introduction

Chronic diseases and behavioral health conditions are among the leading contributors to healthcare utilization and rising healthcare costs in the United States. Approximately six in ten Americans live with at least one chronic condition—such as heart disease, cancer, or diabetes—while nearly one in five adults experience a mental illness annually, and approximately one in twelve adults aged 18 or older have a substance use disorder (SUD) [1–3]. These conditions often require ongoing management, including frequent healthcare visits, medication adjustments, and monitoring, all of which place substantial demand on healthcare systems.

The COVID-19 pandemic profoundly disrupted healthcare delivery and patient outcomes globally, triggering widespread shifts in how and when patients access care. Early evidence indicates that the pandemic interrupted routine care for patients with chronic illnesses, potentially exacerbating existing disparities in healthcare access and quality [4–6]. In the months following COVID-19 infection, patterns of healthcare utilization may change due to a combination of direct viral effects, such as post-acute sequelae, and indirect impacts related to care avoidance, healthcare system strain, or changes in clinical follow-up practices [7, 8]. Patients with pre-existing conditions are particularly vulnerable to these disruptions, as the first six months after infection represent a critical period marked by increased clinical vulnerability and risk for post-acute complications [9, 10].

In this study, pre-existing conditions were selected to represent a broad yet clinically meaningful spectrum of chronic health needs that could be affected by COVID-19 exposure. They represent a mix of high-burden noncommunicable diseases (e.g., diabetes, heart failure, kidney disease, cancer), mental health conditions (e.g., anxiety and depression), and acute events often tied to behavioral health (e.g., drug poisoning/overdose). These conditions are prevalent, clinically significant, and have well-documented patterns of healthcare utilization—making them especially sensitive to disruptions in care delivery. Including thyroid disorders provides insight into the effects of the pandemic on conditions that, while generally less acute, require routine monitoring and long-term management [11, 12]. Together, this set reflects a wide range of physical and mental health challenges, allowing us to evaluate how COVID-19 may have influenced access, engagement, and continuity of care for patients with pre-existing health needs.

### Prior work

Healthcare disparities remain a well-documented challenge in the U.S. healthcare system, disproportionately affecting historically marginalized and socioeconomically disadvantaged populations. Black, Hispanic, and Indigenous communities face higher baseline rates of chronic conditions and are more likely to work in high-exposure occupations, which elevate their risk of infection and hospitalization [13]. Sex-based differences in healthcare access have also been observed, highlighting the need to consider multiple dimensions of disparity in understanding care patterns during the pandemic [14].

The COVID-19 pandemic precipitated substantial declines in overall healthcare utilization, with pronounced decreases in chronic disease management services including diabetes care, heart failure treatment, and cancer chemotherapy [15, 16]. Simultaneously, telemedicine rapidly expanded, reshaping healthcare access and delivery modalities, though its long-term effects on post-COVID care and patient outcomes remain unclear [17].

Patients have disease specific heterogeneous utilization patterns. For example, during the pandemic’s peak, emergency department visits for diabetes-related complications increased, likely due to delayed outpatient care, followed by a return to baseline as telemedicine uptake increased [18–20]. Heart failure hospitalizations declined early in the pandemic, likely reflecting patients’ fear of infection and altered care-seeking behavior [21]. Patients with kidney disease experienced reduced hospital admissions for non-COVID-19 conditions, despite higher rates of acute kidney injury related to COVID-19 infection [22, 23]. Cancer care delivery faced widespread disruptions, including delayed treatments and surgeries, alongside increased use of telehealth for low-risk patients [24, 25]. Thyroid disorders were associated with altered care patterns during the pandemic, likely driven by access barriers and the multifaceted effects of acute COVID-19 infection—both during illness and in the weeks following recovery [26]. Mental health conditions worsened during the pandemic. Anxiety disorders rose by up to 25%, accompanied by increased emergency visits driven by psychological stress, social isolation, and limited outpatient service availability [27–29]. Depression-related primary care visits initially declined but telehealth utilization remained elevated [30]. Substance use disorders were exacerbated, contributing to surges in overdose deaths and challenges in accessing treatment [31].

### Goal of this study

This study aims to evaluate the impact of COVID-19 infection on healthcare utilization among individuals with a range of pre-existing chronic physical and behavioral health conditions—specifically diabetes, heart failure, kidney disease, cancer, thyroid disorders, anxiety, depression, and drug poisoning/overdose—selected for their clinical importance, high prevalence, and known vulnerability during and after the COVID-19 pandemic.

We hypothesize that COVID-19 infection may impact healthcare utilization in multiple ways: it could increase utilization by making individuals more vulnerable to complications from pre-existing conditions (due to heightened illness severity), lead to increased care-seeking as a result of the infection itself, or conversely, decrease utilization due to disruptions in access to care, fear of exposure, or changes in health system operations during the pandemic.

We further seek to understand how demographic characteristics (age, biological sex, race, ethnicity) and ecological socioeconomic indicators (poverty/food security, income, education) derived from patients’ residential U.S. Census tract codes, are associated with differences in healthcare utilization patterns post-infection.

Through this comprehensive approach, we aim to elucidate heterogeneous effects of COVID-19 exposure on healthcare engagement and identify populations at risk for care disruptions, informing targeted interventions to mitigate long-term adverse health outcomes.

## Methods

### Data

#### Clinical data

Patient-level data^1^ were extracted from the Electronic Health Records (EHR) of the MedStar Health Research Institute, which serves a network of 10 hospital systems and affiliated outpatient clinics located in Washington, D.C., Maryland, and Virginia.

Patient demographic characteristics and clinical factors associated with healthcare utilization were examined. Age at time of COVID-19 testing was modeled categorically (18–34, 35–54, 55–64, and ≥65 years). Biological sex was categorized as male or female. Race was classified as Black, White, or Other/Unknown, and ethnicity as Hispanic, Non-Hispanic, or Other/Unknown. Insurance status was categorized into commercial, Medicare, self-pay, government-sponsored, or other/unknown. Obesity status (body mass index ≥ 30) was included as a binary variable given its known associations with increased morbidity and healthcare utilization. State of residence (Washington, D.C., Maryland, or Virginia) was included as a categorical variable to account for regional differences in healthcare systems and policy environments. Patient cohorts were identified using validated ICD-10 diagnostic code lists, as detailed in Supplementary Table 1 based on pre-existing chronic physical conditions—namely diabetes mellitus, cancer, kidney disease, heart failure, hyperthyroidism, and hypothyroidism—as well as behavioral health conditions including anxiety, depression, and drug poisoning and overdose (hereafter collectively referred to as the *study diagnoses*).

Clinical encounter data spanned four distinct care settings: outpatient visits, inpatient hospitalizations, emergency department (ED) visits, and virtual care encounters. Encounter categories were derived by combining structured information from encounter and admission records. *Inpatient* encounters include inpatient and observation stays. *Outpatient* encounters include visits classified as outpatient, home health, or pre-admission. *Emergency* encounters include only those explicitly labeled as emergency encounters including encounters with a corresponding emergency admission. *Virtual* encounters include telehealth visits and secure inbox messages. Encounters with incomplete or conflicting data were excluded.

Utilization was assessed over three time periods as described in Sect. 2.2 and further examined by the study diagnoses.

#### Ecological socioeconomic indicators

Socioeconomic determinants of health were assessed using ecological indicators at the U.S. Census tract level from the 2020 American Community Survey (ACS). These included inflation-adjusted median household income, the percentage of adults aged 25 and older holding advanced degrees (master’s, professional, or doctoral), and the proportion of households receiving Supplemental Nutrition Assistance Program (SNAP) benefits below the federal poverty line. Individual patients were linked to these indicators via residential addresses mapped to corresponding Census tracts.

To facilitate standardized regional comparisons, each socioeconomic variable was divided into tertiles—Low, Medium, and High—within each state, capturing relative socioeconomic status (SES) in local contexts and emphasizing geographic disparities. Descriptions of these variables appear in Supplementary Table 2, and their spatial distributions across Washington, D.C., Maryland, and Virginia are illustrated in Supplementary Figure 1.

### Study cohort and periods

The study population comprised adult patients (≥18 years) with at least one healthcare encounter within the MedStar Health system between March 25, 2019, and June 30, 2021, who had at least one pre-existing study condition before the testing period. Patients who tested positive for COVID-19 between March 11, 2020, and December 17, 2020, were designated as the exposure group, while those testing negative for COVID-19 during the same timeframe comprised the control group.

We adopted a six-month observation window—a timeframe commonly used in prior research to evaluate changes in healthcare utilization, including increases in healthcare encounters, emergency department visits, and the impact of multidisciplinary post-COVID care on outcomes [32–34]. This approach enables the examination of utilization trends relative to COVID-19 infection and supports the identification of changes potentially attributable to the infection itself.

**Fig. 1:**
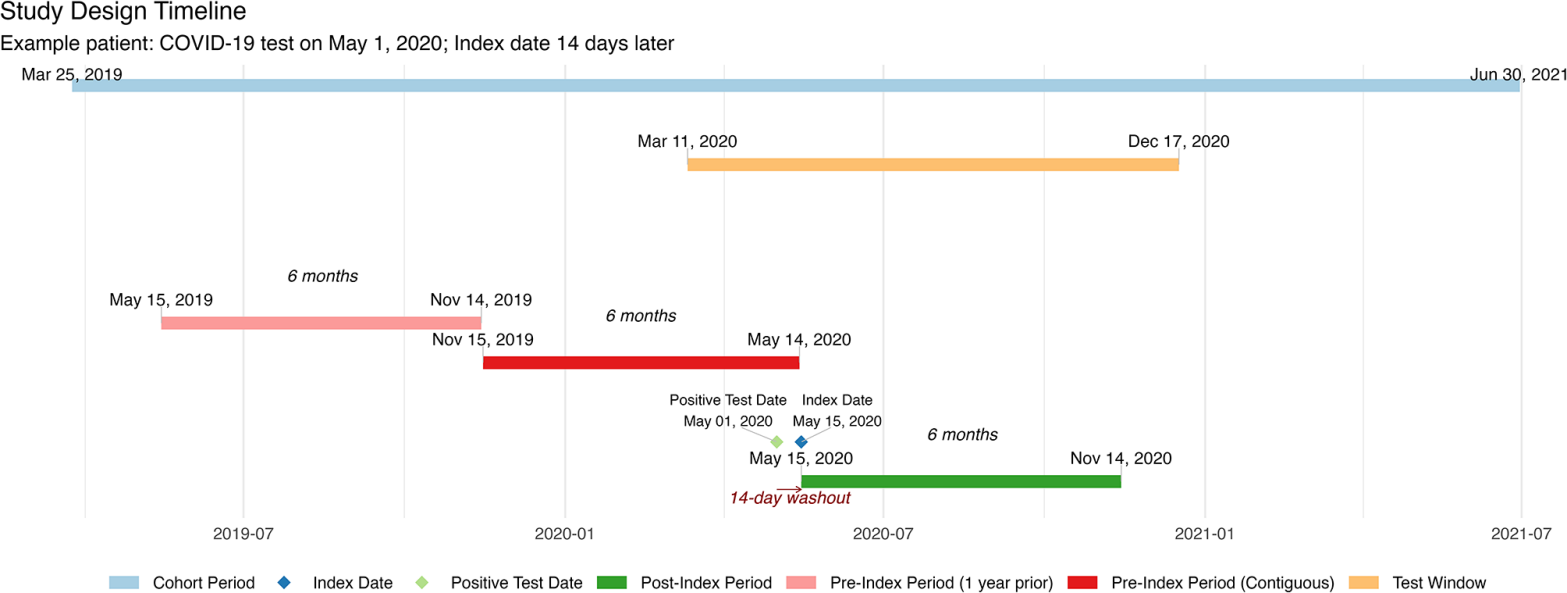
Study design timeline illustrating key periods relative to an example patient’s COVID-19 test date (May 1, 2020). The timeline shows the cohort period, test window, the two pre-index periods (*baseline* [one year prior] and *contiguous* 6 months), and the post-index period. Points mark the positive test date and the index date (14 days later, representing a 14-day washout period)

The index date was defined as 14 days following the date of the COVID-19 test result (positive or negative), allowing for a two-week washout period to reduce confounding from acute illness episodes. The post-index period encompassed the six months following this date. We evaluated healthcare utilization across three time periods: (1) the *post-index period,* a six-month window after the index date; (2) the *contiguous pre-index period*, the six months immediately preceding the index date; and (3) the *baseline pre-period*, a six-month window from the same calendar months one year prior to the post-index period. Figure 1 illustrates this timeline, highlighting key periods including the cohort period, testing window, index date, and pre- and post-index periods. For example, a patient testing positive on May 1, 2020, had an index date of May 15, 2020; their post-index period extending from May 15, 2020, to November 14, 2020. The first pre-index period was from May 15 to November 14, 2019, and the second pre-index period was from November 15, 2019, to May 14, 2020.

We then constructed a matched retrospective cohort. Exposure group patients were matched 1:1 with controls within the same pre-existing disease group, based on exact matching for biological sex and state of residence, and nearest neighbor propensity score matching for age (±5 years), test month/year, obesity status (body mass index ≥ 30), race (categorized as Black, White, or Multiple/Other/Unknown), and ethnicity (Hispanic, Non-Hispanic, Multiple/Other/Unknown). These matching variables were selected due to their documented associations with healthcare utilization and COVID-19 outcomes. When multiple control candidates met matching criteria, the control with the closest propensity score within the caliper was selected. The same observation periods were applied to the matched controls.

Patients who died during the study period were excluded because their health care utilization patterns would not be comparable over the full follow-up window, potentially biasing estimates of post-COVID health care use. We also excluded patients who resided outside of Washington, D.C., Maryland, or Virginia to ensure consistent geographic data coverage, particularly for socioeconomic indicators derived from Census tract data, and to minimize variability in health system access and reporting across different regions. To ensure completeness of data and active healthcare engagement, patients were required to have at least one healthcare encounter both before the first pre-index period and after the post-index period.

### Statistical analysis

Baseline demographic and clinical characteristics were summarized using descriptive statistics and compared between COVID-19 positive (exposed) and negative (control) groups to assess post-matching balance. Further, weekly utilization trends were assessed by fitting simple linear regression models to weekly utilization observations across the baseline pre vs. post, and contiguous pre vs. post periods.

To estimate the effect of COVID-19 infection on healthcare utilization, we applied a difference-in-differences (DiD) analytical framework [35] via Poisson regression [33]. Poisson models were chosen for their appropriateness in modeling count data and their widespread application in health services research. The primary outcome was healthcare utilization, measured as the number of unique clinical encounters per patient.

Each model included main effects for COVID-19 case status and time period (*baseline/contiguous* pre- vs. post-index), along with an interaction term (post × case), which served as the DiD estimator. This approach adjusts for individual-level characteristics that remain constant over time, such as baseline health status and care-seeking behaviors, because their effects cancel out when comparing pre-to-post changes between groups. The contemporaneous control group accounts for broader pandemic-related disruptions in healthcare delivery. Statistical significance of the interaction term was evaluated using Wald tests, with two-sided *p*-values < 0.05 considered statistically significant and *p*-values between 0.05 and 0.10 considered marginally significant.

We estimated overall DiD rate ratios (RR) for healthcare utilization and, to examine variation across populations and conditions, calculated stratum-specific DiD RRs for subgroups defined by visit type, age, sex, race, ethnicity, insurance type, obesity status, state, neighborhood-level socioeconomic indicators (income, education, SNAP participation), and study disease. Separate DiD models were fit for each stratum–disease combination, producing subgroup-specific estimates with 95% confidence intervals and *p*-values, allowing assessment of how the intervention’s impact varied across different populations and conditions.

To facilitate interpretation of the rate ratios, we also computed absolute changes in healthcare use, translating model estimates into the number of excess (or reduced) visits per 1,000 COVID-19–positive patients using observed post-index encounter volumes.

All analyses were conducted in R software [36]. Further methodological details, including model specifications and derivation of excess healthcare visits attributable to COVID-19 exposure, are provided in Supplementary Appendix A.

## Results

### Cohort characteristics

Patients counts before matching are detailed in Supplementary Table 3.

Demographic and socioeconomic characteristics of the matched cohorts by pre-existing conditions are provided in Table 1, comprising 8,653 COVID-19–positive cases and 13,205 negative controls across all study disease groups, with generally good balance observed between groups.

**Table 1:** Overall counts and percentages by demographic, socioeconomic, and clinical characteristics of COVID-19 positive (cases) and negative (controls) cohorts matched 1:1 by condition. Conditions are not mutually exclusive—patients may appear in more than one column if they had multiple conditions

	Anxiety	Depressive Disorders	Diabetes Mellitus	Drug Poisoning / Overdose	Heart Failure	Hyperthyroidism	Hypothyroidism	Kidney Disease	Malignant Neoplasms	All Study Disease Groups	
	Case / Control	Case / Control	Case / Control	Case / Control	Case / Control	Case / Control	Case / Control	Case / Control	Case / Control	Case	Control
**Total patients**	4,414 / 4,414	2,786 / 2,786	4,921 / 4,921	1,732 / 1,732	1,529 / 1,529	266 / 266	1,534 / 1,534	2,939 / 2,939	1,447 / 1,447	8,653	13,205
**Test Month**											
… 2020–03	0.2% / 0.2%	0.2% / 0.1%	0.5% / 21 0.4%	0.2% / 0.2%	0.5% / 0.5%	0.4% / 0.4%	0.1% / 0.1%	0.6% / 0.4%	0.1% / 0.1%	0.3%	0.3%
… 2020–04	10.4% / 10.1%	11.2% / 10.2%	14.7% / 8.7%	13.7% / 9.8%	15.3% / 8.8%	12.4% / 6.8%	12.5% / 9.3%	17.1% / 8.9%	12.2% / 9.9%	11.7%	6.6%
… 2020–05	11.6% / 11.8%	13.1% / 12.7%	16% / 14.7%	14.6% / 14.5%	15.6% / 16.8%	14.3% / 12.8%	13.2% / 13.7%	16.7% / 15.5%	14.4% / 14.2%	13.4%	10.7%
… 2020–06	7.4% / 6.8%	8% / 7.8%	8% / 7.3%	7% / 5.6%	6.9% / 6.6%	4.9% / 3.8%	7.2% / 6.7%	7.7% / 7.3%	8.2% / 7.7%	7.7%	7.8%
… 2020–07	11.9% / 11.9%	10.5% / 10.4%	8.7% / 8.8%	7.3% / 7.8%	7.5% / 106.9%	13.2% / 13.9%	9.8% / 9.8%	7.6% / 7.6%	9.5% / 8.8%	10.3%	11.1%
… 2020–08	9.3% / 9.5%	9.7% / 9.8%	8.2% / 9%	8.1% / 8.9%	8.6% / 138.6%	7.9% / 7.5%	9.1% / 9.6%	7.7% / 8.3%	8.8% / 9.5%	9.2%	10.7%
… 2020–09	6.2% / 6.1%	6% / 6%	5.1% / 5.3%	6.6% / 5.9%	5.6% / 6%	6.4% / 6.4%	4.8% / 4.3%	5% / 4.7%	5.2% / 5%	6%	6.8%
… 2020–10	9% / 9%	8.9% / 8.9%	7.6% / 7.2%	9.3% / 9.4%	8.6% / 8.5%	8.6% / 9.8%	7.8% / 7.2%	7.3% / 7%	8% / 7.7%	8.8%	9.8%
… 2020–11	19.6% / 19.3%	17.8% / 18%	16.7% / 19.5%	18.3% / 19.5%	15.4% / 17.1%	17.7% / 22.2%	19.6% / 20.7%	16% / 19.3%	17.3% / 18.8%	17.8%	20%
… 2020–12	14.5% / 15.3%	14.5% / 16.2%	14.5% / 19.1%	14.8% / 18.2%	15.9% / 20.1%	14.3% / 16.5%	16% / 18.5%	14.4% / 21%	16.2% / 18.3%	14.7%	16%
**Age**											
… 18–34	23.7% / 23.6%	20.2% / 20.1%	4.6% / 4.8%	10.3% / 9.8%	2.2% / 2.5%	11.3% / 13.2%	6.6% / 6.9%	2.9% / 2.9%	4.2% / 3.9%	15%	12.8%
… 35–54	35.1% / 34.8%	30.9% / 31%	27.6% / 26.8%	26.4% / 26.5%	15% / 15.6%	28.2% / 26.7%	24.1% / 24%	17.7% / 17.8%	18.3% / 18.9%	30.4%	28.4%
… 55–64	18.4% / 19.1%	19.2% / 19.9%	26.4% / 27.3%	22.3% / 22.9%	21.4% / 21.1%	18% / 22.9%	21.8% / 22.4%	20.7% / 21.7%	21.8% / 22.6%	21.2%	22.2%
… 65+	22.8% / 22.5%	29.7% / 28.9%	41.5% / 41.1%	41% / 40.8%	61.5% / 60.8%	42.5% / 37.2%	47.5% / 46.7%	58.7% / 57.7%	55.6% / 54.6%	33.3%	36.5%
**Sex**											
… Female	69.5% / 69.5%	69.2% / 69.2%	54.5% / 54.5%	54.3% / 54.3%	49.8% / 49.8%	77.4% / 77.4%	75.6% / 75.6%	49.7% / 49.7%	55.9% / 55.9%	62.2%	61.4%
… Male	30.5% / 30.5%	30.8% / 30.8%	45.5% / 45.5%	45.7% / 45.7%	50.2% / 50.2%	22.6% / 22.6%	24.4% / 24.4%	50.3% / 50.3%	44.1% / 44.1%	37.8%	38.6%
**Race**											
… White	44.4% / 45.6%	46.6% / 48.1%	60.4% / 62.1%	59.9% / 61.4%	64.2% / 63.3%	63.9% / 61.7%	38.3% / 38.7%	66.2% / 65.7%	39.5% / 41%	33.7%	36.5%
… Black	13.2% / 10.9%	11.1% / 8.4%	17.8% / 13%	13.2% / 10%	9% / 7.7%	9.8% / 10.5%	14.8% / 11.7%	9.8% / 7.1%	48.4% / 50.1%	52.2%	53.2%
… Multiple/Other/Unknown	42.3% / 43.5%	42.3% / 43.4%	21.8% / 24.9%	26.9% / 28.6%	26.9% / 29%	26.3% / 27.8%	46.9% / 49.5%	24% / 27.3%	12% / 8.9%	14.1%	10.3%
**Ethnicity**											
… Hispanic	4% / 2.8%	3.9% / 2.7%	5.3% / 4%	4.6% / 3.2%	2.3% / 2.2%	1.5% / 0.4%	5.1% / 4.2%	2.7% / 2%	4.4% / 3.2%	4.4%	3%
… Non-Hispanic	6% / 4.7%	5.3% / 3.9%	9.7% / 7.2%	6.8% / 5.2%	5.6% / 4.1%	3% / 3.8%	7% / 4.6%	6.1% / 4.2%	90.3% / 93.4%	88.8%	92.2%
… Multiple/Other/Unknown	90.1% / 92.5%	90.8% / 93.4%	84.9% / 88.8%	88.7% / 91.6%	92.2% / 93.8%	95.5% / 95.9%	87.9% / 91.3%	91.2% / 93.7%	5.4% / 3.3%	6.9%	4.8%
**Obese**	48.3% / 47.6%	50% / 50.7%	59.2% / 59.4%	49.5% / 48.2%	51.5% / 51.4%	51.5% / 46.6%	49.2% / 49.5%	46.4% / 46.2%	43.7% / 42.6%	51.4%	51%
**U.S. State of Residence**											
… DISTRICT OF COLUMBIA	20.9% / 20.9%	20.3% / 20.3%	22.6% / 22.6%	24.4% / 24.4%	26.1% / 26.1%	23.3% / 23.3%	17.8% / 17.8%	27.7% / 27.7%	23.3% / 23.3%	23%	22%
… MARYLAND	75.9% / 75.9%	77.4% / 77.4%	75.4% / 75.4%	74.4% / 74.4%	72.6% / 72.6%	75.6% / 75.6%	79.5% / 79.5%	70.7% / 70.7%	73.6% / 73.6%	74.2%	75.2%
… VIRGINIA	3.1% / 3.1%	2.3% / 2.3%	2.1% / 2.1%	1.3% / 1.3%	1.3% / 1.3%	1.1% / 1.1%	2.7% / 2.7%	1.7% / 1.7%	3.1% / 3.1%	2.8%	2.8%
**Household Income Level**											
… Low	57.7% / 60.7%	51.9% / 55.6%	50.2% / 53.9%	49% / 50.3%	47.6% / 53.2%	52.3% / 57.9%	58.3% / 64.1%	48.3% / 52.3%	14% / 11.8%	16.3%	15.5%
… Medium	14.5% / 13.9%	16.6% / 15.7%	18.6% / 18.6%	19.6% / 18.4%	20.1% / 18.6%	19.5% / 15.4%	14.1% / 10.3%	19.8% / 19%	26.1% / 24.5%	28.6%	26.2%
… High	27.8% / 25.4%	31.5% / 28.7%	31.3% / 27.5%	31.4% / 31.2%	32.3% / 28.2%	28.2% / 26.7%	27.5% / 25.6%	31.9% / 28.7%	60% / 63.7%	55%	58.4%
**Graduate / Professional Degree (%)**											
… Low	53.7% / 54.3%	49.5% / 49.5%	47.2% / 48.2%	48.2% / 47.5%	45.1% / 48.3%	48.1% / 53.8%	51.8% / 56.6%	47.2% / 49.1%	18.7% / 12.8%	17.7%	15.6%
… Medium	17.1% / 15.7%	19.3% / 17.1%	20% / 17.3%	19.9% / 18.4%	21.1% / 17.5%	18% / 14.3%	18.4% / 13.2%	19.7% / 16.6%	27% / 30.4%	30.5%	32.5%
… High	29.1% / 30%	31.2% / 33.4%	32.8% / 34.4%	31.9% / 34.1%	33.8% / 34.1%	33.8% / 32%	29.9% / 30.2%	33.1% / 34.4%	54.4% / 56.8%	51.8%	51.9%
**Households on Food Stamps / SNAP (%)**											
… Low	28.1% / 26.1%	31.7% / 28.3%	33.2% / 31.2%	34.3% / 32.2%	34.6% / 33.6%	34.6% / 30.5%	26.9% / 21.3%	35.1% / 32.9%	37.9% / 40.5%	33.8%	36%
… Medium	34.9% / 37%	30.8% / 34.1%	33.1% / 32.7%	30.2% / 30.3%	29.1% 30.7%	32.7% / 34.2%	36% / 39.2%	30.8% / 32.3%	35.2% / 35.8%	36%	36.5%
… High	37% / 36.9%	37.5% / 37.6%	33.8% / 36.2%	35.5% / 37.6%	36.3% / 35.7%	32.7% / 35.3%	37.1% / 39.6%	34.2% / 34.8%	26.8% / 23.7%	30.2%	27.5%

Testing patterns were broadly consistent across study disease subgroups, with peaks observed in the later months of 2020, reflecting temporal trends in testing availability and pandemic progression. Age distributions reflected the expected epidemiology of each condition and were similar between COVID-19 positive and negative patients within each disease group. Similar age patterns were observed for conditions such as kidney disease (59% cases aged 65+) malignant neoplasms (56% aged 65+), diabetes mellitus (42% aged 65+), and heart failure (about 62% aged 65+). For Anxiety Disorders, most patients were aged 35–54 (35%), followed by 18–34 (24%), with 23% of cases aged 65+. Depressive Disorders had a similar pattern, with 35–54 making up about 31%, 18–34 around 20%, and 65+ nearly 30%. For Drug Poisoning/Overdose, older adults (65+) were the largest group (41%), while 35–54 accounted for about 26%, and 18–34 roughly 10%. For Hyperthyroidism, patients were more evenly distributed across age groups, with the highest proportions in 35–54 (28.2% cases) and 65+ (42.5% cases). For Hypothyroidism, nearly half were aged 65+ (47.5% cases), followed by 35–54 (24.1% cases), indicating these conditions are most common in middle-aged and older adults. Sex distribution was generally balanced in kidney disease, malignant neoplasms, diabetes, heart failure, and drug overdose. In contrast, females comprised a clear majority in hyperthyroidism, hypothyroidism, anxiety, and depressive disorders, accounting for approximately 69–77%. Racial composition showed variation across disease groups. White patients made up the majority in kidney disease (66%), diabetes (60%), heart failure (64%), hyperthyroidism (64%), and drug poisoning/overdose (60%). They were slightly less represented in anxiety (45%), depressive disorders (47%), malignant neoplasms (40%) and hypothyroidism (38%). Black patients comprised notable proportions in malignant neoplasms (49%) and diabetes (18%), with lower representation in other conditions. Ethnicity was predominantly categorized as Multiple/Other/Unknown (over 85% in most groups) or non-Hispanic. Hispanic patients accounted for a small proportion across diseases, with the highest observed in diabetes mellitus (5%), hypothyroidism (5%), and drug poisoning/overdose (5%). Obesity prevalence was close to 50% in most study diseases and exceeded 50% in a few, reaching the highest level in diabetes (59%). Socioeconomic patterns differed by condition. A majority of patients with kidney disease (48%), diabetes mellitus (50%), heart failure (48%) hyperthyroidism (52%), and hypothyroidism (58%) were from low-income households. Low-income representation was also high in anxiety disorders (58%), depressive disorders (52%), and drug poisoning/overdose (49%). In contrast, patients with malignant neoplasms were more often from high-income households (60%). Educational attainment followed a similar pattern. A large share of patients with malignant neoplasms had high levels of graduate or professional education (55%), whereas lower educational attainment of about 50% was more common in patients with kidney disease, diabetes, heart failure, thyroid conditions, and mental health or drug poisoning/overdose. Poverty-related food insecurity also differed across conditions. Over one-third of patients with kidney disease (35%), diabetes (33%), and drug poisoning/overdose (34%) lived in neighborhoods with low SNAP participation. Meanwhile, high SNAP usage was slightly more common in depressive disorders (38%), anxiety (37%), thyroid conditions (37%), hearth failure (36%), and drug poisoning/overdose (36%).

### Healthcare encounter characteristics

Table 2 presents encounter counts for COVID-19-positive cases and matched controls across the two pre-index periods and the post-index period, stratified by condition. A consistent temporal pattern in healthcare utilization emerged around the time of COVID-19 testing. Encounter volumes increased sharply during the contiguous pre-period compared to the baseline pre-period (1 year before), followed by a return to baseline or a slight decline in the post- period. This pattern suggests a transient intensification of healthcare use surrounding the COVID-19 testing window.

**Table 2:** Healthcare encounter counts for matched patients stratified by disease category and the 6-months study periods (baseline pre-, contiguous pre- and post-periods)

	Pre-Index Period (1 year prior)	Pre-Index Period (Contiguous)	Post-Index Period
	**Case / Control**	**Case / Control**	**Case / Control**
Anxiety Disorders	19,033 / 21,284	32,324 / 34,944	29,398 / 32,072
Depressive Disorders	14,092 / 15,233	21,944 / 24,180	20,058 / 22,029
Diabetes Mellitus	18,540 / 23,273	29,795 / 38,734	25,789 / 35,435
Drug Poisoning / Overdose	8,788 / 10,815	13,062 / 17,126	12,742 / 17,648
Heart Failure	8,284 / 9,502	12,085 / 14,695	11,765 / 14,410
Hyperthyroidism	1,435 / 1,839	2,276 / 2,615	2,131 / 2,440
Hypothyroidism	7,193 / 7,683	11,299 / 12,922	10,038 / 11,836
Kidney Disease	14,277 / 17,260	21,415 / 27,500	19,356 / 27,385
Malignant Neoplasms	8,032 / 9,205	12,465 / 15,242	12,059 / 14,373

**Fig. 2:**
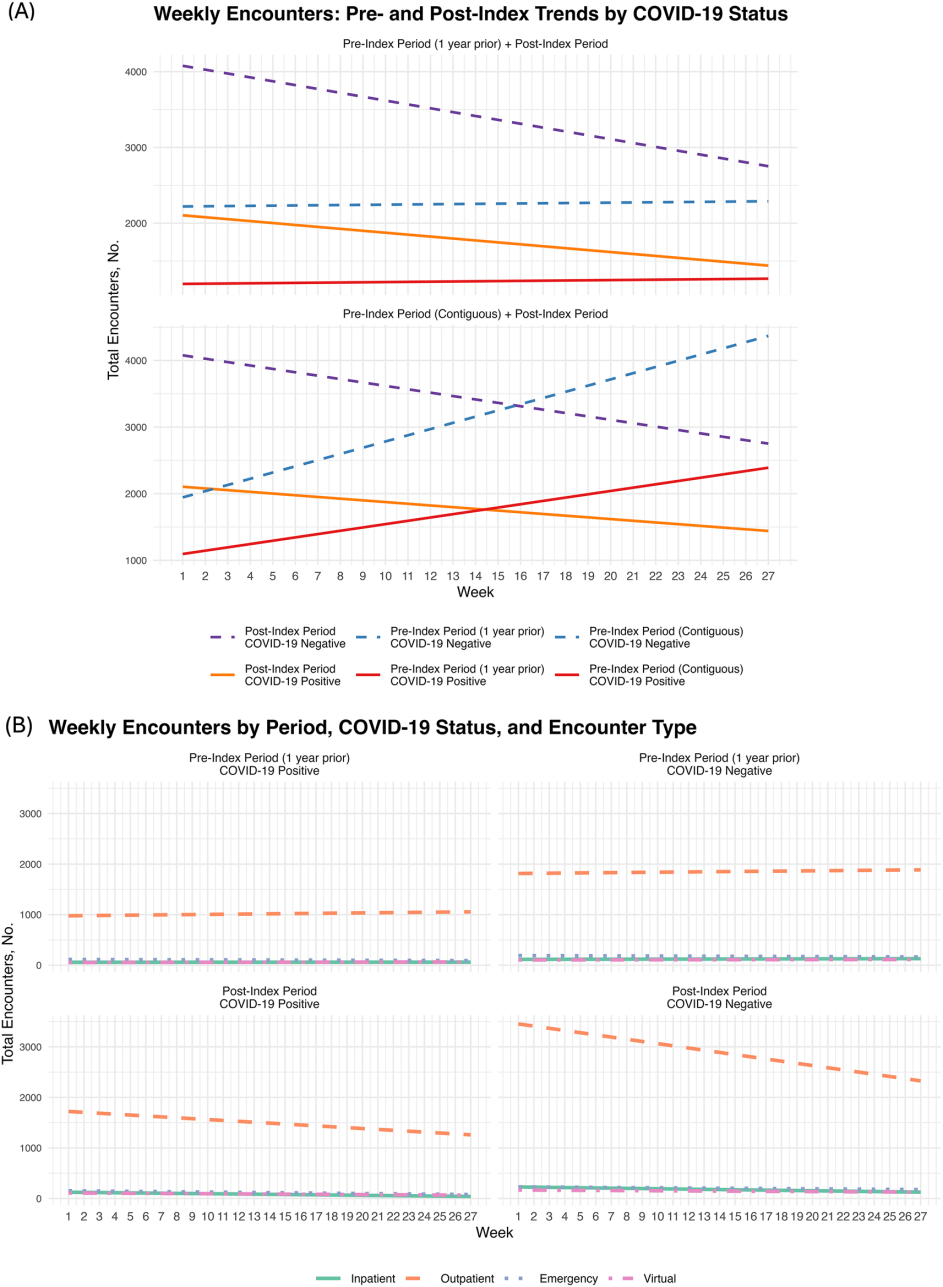
Trends in healthcare utilization over time (weeks in pre-index [baseline and contiguous] vs post-index period) among matched case-control cohorts, illustrating baseline comparability and temporal patterns overall (**A**) and by encounter type (**B**); a regression line was fitted to weekly encounter counts to visualize overall trends

Supplementary Table 4 provides further stratification by encounter type and insurance category. Across all conditions, a similar temporal shift was observed, with a peak in encounters during the contiguous pre-period, followed by a decrease in the post-period that nevertheless remained elevated relative to the baseline pre-period. Outpatient care comprised the majority of encounters across all time points and conditions; however, the relative proportion of outpatient visits declined during the contiguous pre-period. For example, among COVID-19-positive patients with pre-existing diabetes, outpatient visits decreased from 81.1% in the baseline pre-period to 66.1% in the contiguous pre-period, with a subsequent rebound to 76.5% in the post-period, below baseline. This decrease in outpatient care was accompanied by an increase in inpatient encounters, which rose from 4.9% to 13.1% during the contiguous pre-period before declining to 4.7% post-period. Virtual care usage also increased substantially across all conditions during and after the index date. Among diabetes patients, virtual encounters increased from 6.6% (baseline pre-period) to 11.9% (contiguous pre-period) and further to 13.6% post-period. Similar trends for virtual care were observed in anxiety disorders (8.8% / 16.0% / 17.5%), depressive disorders (7.7% / 14.9% / 17.3%), and hypothyroidism (7.3% / 13.4% / 13.7%). These findings suggest that the period surrounding COVID-19 testing was marked by acute care intensification—reflected by inpatient and emergency care use—and accelerated adoption of virtual care, which remained elevated in the post-period, indicating potential long-term shifts in care delivery. Insurance patterns showed relative stability across periods, with traditional public and private coverage remaining predominant. Commercial insurance accounted for 20–29% of encounters across conditions and time points, showing minimal variation. Medicare coverage was highest among patients with cardiometabolic and endocrine conditions—including 47.5% of the heart failure and 45.8% of the kidney disease cohorts during the baseline pre-period—reflecting their older age profile. However, the Medicare’s share modestly declined during the baseline pre-period to the contiguous pre-period and remained slightly reduced in the post-period. Unspecified or “Other/Unknown” insurance categories saw modest increases around the testing date (contiguous pre-period and post-period compared to baseline pre-period), potentially indicating temporary disruptions in coverage or administrative delays. Self-pay and Veteran/Amerigroup/Other Government coverage remained consistently low (<3% for most conditions), though with slight upticks during the baseline pre-period, likely reflecting urgent care needs or lapses in traditional coverage. Figure 2 illustrates healthcare utilization for exposure cases and controls during the baseline pre-period, contiguous pre-period, and post-period, with regression lines fitted to weekly encounters. Utilization trends during the baseline pre-period were parallel between exposure and control groups, supporting baseline comparability and justifying its use as the reference period for subsequent difference-in-differences (DiD) analyses. No meaningful divergence in utilization was observed overall (Fig. 2A) or by encounter type (Fig. 2B), further supporting the parallel trends assumption. This pattern was consistent across study disease subgroups with pre-existing conditions (Supplementary Figures 2 and 3), reinforcing the validity of the DiD approach—i.e., in the absence of COVID-19 exposure, outcomes in the case and control groups would have been expected to follow similar trajectories over time.

**Fig. 3:**
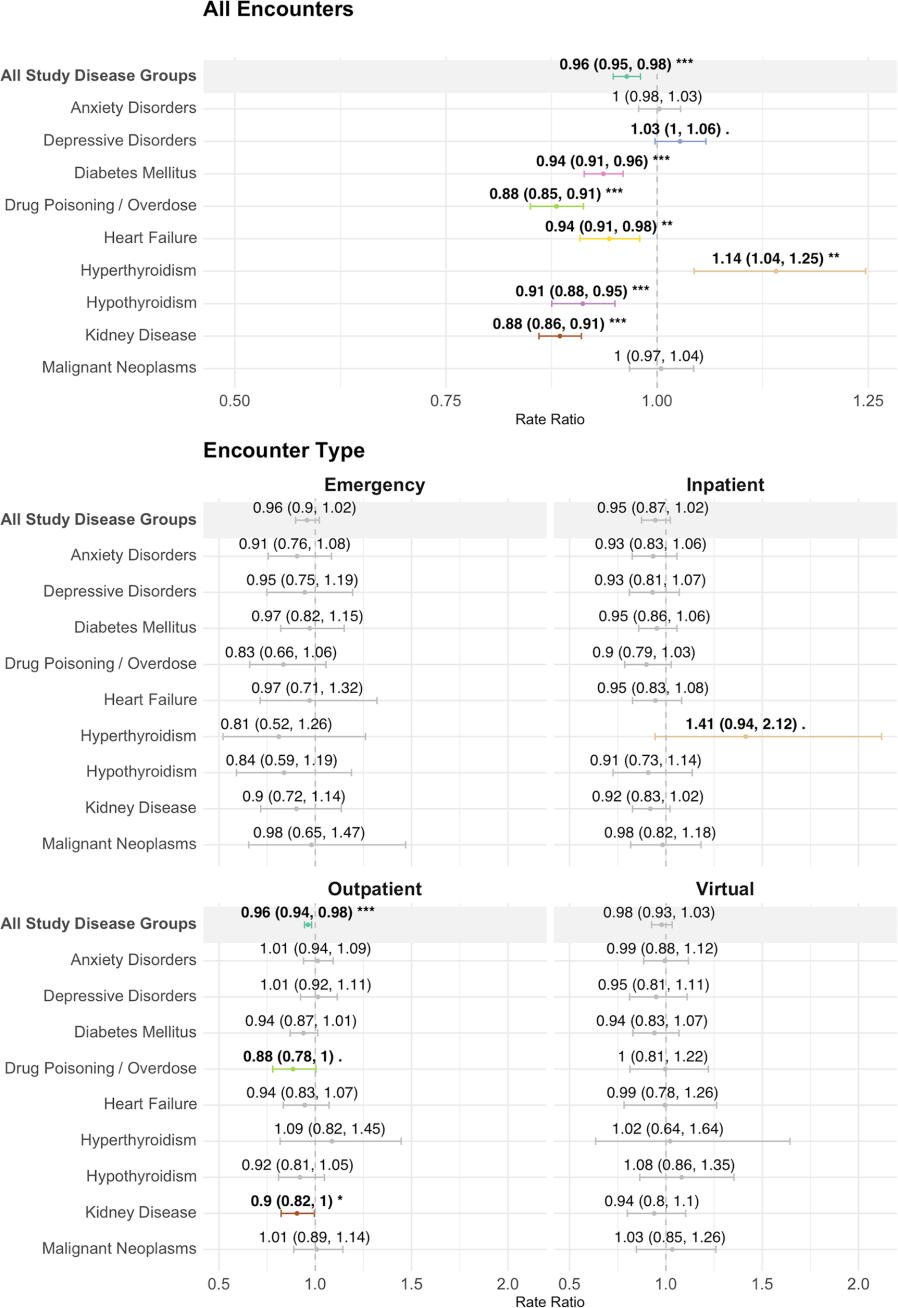
Healthcare utilization associated with positive versus negative COVID-19 test results. The difference-in-differences estimate parameter represents the adjusted change in encounter rates comparing post- versus pre-periods, estimated using Poisson regression models. Significance codes: “***” p ≤ 0.001; “**” p ≤ 0.01; “*” p ≤ 0.05; “.” p ≤ 0.10. Colors indicate disease categories only for statistically significant estimates in bold (p ≤ 0.10).

### Difference-in-differences analysis of COVID-19 impact on healthcare utilization

Using Poisson-based difference-in-differences (DiD) models, we evaluated the impact of COVID-19 infection on healthcare utilization both overall and by study disease, stratified across visit types, demographic characteristics, and socioeconomic factors. Rate ratios (RRs) were calculated to compare changes in utilization between the post- and pre-periods for cases versus matched controls. An RR of 1.0 indicates no change in utilization, values below 1.0 indicate reduced utilization, and values above 1.0 indicate increased utilization attributable to COVID-19 exposure. 

Figure 3 displays adjusted RRs with 95% confidence intervals comparing healthcare encounter rates across encounter types (all encounters, emergency, inpatient, outpatient, and virtual) for all study disease groups and selected conditions. 

Healthcare utilization following COVID-19 infection was associated with a modest but statistically significant 4% reduction in total healthcare encounters (RR = 0.96, *p* < 0.001). Several chronic conditions experienced substantially larger declines than the overall estimate, including kidney disease (RR = 0.88, *p* < 0.001), diabetes (RR = 0.94, *p* < 0.001), heart failure (RR = 0.94, *p* = 0.002), and hypothyroidism (RR = 0.91, *p* < 0.001). Encounters for drug poisoning/overdose also declined markedly (RR = 0.88, *p* < 0.001). In contrast, hyperthyroidism was the only condition associated with a significant increase in utilization (RR = 1.14, *p* = 0.003), while depressive disorders exhibited only a marginal rise (RR = 1.03, *p* = 0.073). Further stratification by visit type showed that the overall 4% reduction was largely driven by declines in outpatient visits (RR = 0.96, *p* < 0.001), whereas changes in inpatient, emergency, and virtual visits were not statistically significant at the population level. Outpatient encounters declined most notably for kidney disease (RR = 0.90, *p* = 0.040) and marginally for drug poisoning/overdose (RR = 0.88, *p* = 0.058). By contrast, the increase observed for hyperthyroidism was concentrated in inpatient utilization (RR = 1.41, *p* = 0.093), suggesting that elevated encounters for this condition may reflect more acute or severe presentations. 

Supplementary Figure 4 shows adjusted RRs with 95% confidence intervals for healthcare encounter rates across all study disease groups and specific conditions, stratified by age group, sex, obesity status, race, ethnicity, insurance type, state, and area-level socioeconomic indicators (household income level, percent of residents with a graduate/professional degree, and percent of households receiving food stamps/SNAP). Regarding age, adults aged ≥ 65 years experienced an overall decline in healthcare utilization (RR = 0.92, *p* < 0.001), with disease-specific analyses revealing particularly strong reductions in diabetes encounters (RR = 0.89, *p* = 0.038). Additional age-stratified effects were evident among younger adults: individuals aged 35–54 years showed marked reductions in kidney disease encounters (RR = 0.78, *p* = 0.036), while adults aged 18–34 years exhibited a marginal decline in heart failure–related encounters (RR = 0.50, *p* = 0.090). 

Regarding sex, males experienced a 10% overall decline in healthcare utilization (RR = 0.90, *p* < 0.001). Disease-stratified analyses indicated that this average effect was disproportionately driven by steep reductions in drug poisoning/overdose (RR = 0.81, *p* = 0.029), kidney disease (RR = 0.84, *p* = 0.012), and, to a lesser extent, diabetes (RR = 0.90, *p* = 0.078). In contrast, no consistent or statistically significant disease-level patterns were observed among females. 

Analyses by obesity status showed that individuals with obesity experienced an overall reduction in healthcare utilization (RR = 0.94, *p* < 0.001). Disease-specific patterns revealed even more pronounced declines among obese individuals with diabetes (RR = 0.91, *p* = 0.036) and those with a history of drug poisoning/overdose (RR = 0.83, *p* = 0.030). Substantial reductions were also observed for obese individuals with kidney disease (RR = 0.84, *p* = 0.005). 

Demographic analyses by race demonstrated overall greater declines in utilization among Black individuals (RR = 0.96, *p* < 0.001) and those identifying as Multiple/Other/Unknown race (RR = 0.92, *p* = 0.003). Disease-specific analyses showed notable reductions for Black individuals with diabetes (RR = 0.92, *p* = 0.083). Among individuals of Multiple/Other/Unknown race, declines were observed for drug poisoning/overdose (RR = 0.64, *p* = 0.02), and kidney disease (RR = 0.7, *p* = 0.034), and marginally for heart failure (RR = 0.69, *p* = 0.094). 

Demographic analyses by ethnicity revealed overall reductions for non-Hispanic individuals (RR = 0.97, *p* < 0.001) and those of Multiple/Other/Unknown ethnicity (RR = 0.93, *p* = 0.050). Disease-specific results indicated stronger declines for non-Hispanic individuals with kidney disease (RR = 0.89, *p* = 0.012) and substantial decreases among individuals of Multiple/Other/Unknown ethnicity for drug poisoning/overdose (RR = 0.45, *p* = 0.002) and kidney disease (RR = 0.68, *p* = 0.078). 

Analyses by insurance status showed that overall, only Medicare beneficiaries experienced a clear decrease in healthcare utilization (RR = 0.91, *p* < 0.001), with a smaller decline observed among individuals with Other/Unknown insurance (RR = 0.97, *p* = 0.05). However, several disease-specific patterns diverged from these aggregate findings. Uninsured (Self-Pay) individuals showed no meaningful change in overall utilization, yet exhibited large increases in encounters for heart failure (RR = 2.38, *p* = 0.039), malignant neoplasms (RR = 2.29, *p* = 0.073), kidney disease (RR = 1.95, *p* = 0.053), and hyperthyroidism (RR = 1.64, *p* = 0.002), suggesting a shift toward higher-acuity needs among those without insurance. In contrast, declines were evident for kidney disease among Medicare beneficiaries (RR = 0.86, *p* = 0.017) and commercially insured individuals (RR = 0.85, *p* = 0.070). 

Geographic analyses revealed reductions in healthcare utilization in Washington, DC (RR = 0.93, *p* < 0.001) and Maryland (RR = 0.98, *p* = 0.023), whereas no significant trend was observed in Virginia. Disease-specific patterns in Maryland showed declines exceeding the overall state-level effect, including for drug poisoning/overdose (RR = 0.85, *p* = 0.026) and kidney disease (RR = 0.87, *p* = 0.017). 

Analyses by neighborhood household income level showed that overall healthcare utilization declined across all income strata. Individuals in low-income areas experienced a significant reduction in total encounters (RR = 0.94, *p* = 0.003), as did those in medium-income areas (RR = 0.96, *p* = 0.009) and high-income areas (RR = 0.97, *p* = 0.027). Disease-specific analyses within the high-income group revealed that reductions were most pronounced for kidney disease (RR = 0.85, *p* = 0.018), with marginal declines observed for drug poisoning/overdose (RR = 0.85, *p* = 0.062) and hypothyroidism (RR = 0.86, *p* = 0.088). No other disease-specific estimates reached marginal or statistical significance. 

Analyses by neighborhood educational attainment, measured as the percentage of residents with a graduate or professional degree, showed overall reductions in healthcare utilization across all education strata. Individuals residing in low-education areas experienced a significant decline in total encounters (RR = 0.95, *p* = 0.011), as did those in medium-education areas (RR = 0.97, *p* = 0.026) and high-education areas (RR = 0.97, *p* = 0.005). Disease-specific analyses revealed marginal reductions in selected conditions: in low-education areas, declines were observed for diabetes mellitus (RR = 0.86, *p* = 0.076) and drug poisoning/overdose (RR = 0.79, *p* = 0.077), whereas in medium-education areas, kidney disease showed a marginal decline (RR = 0.87, *p* = 0.072). 

Analyses by neighborhood-level SNAP enrollment showed that overall healthcare utilization of residents of high-SNAP areas experienced a significant reduction in total encounters (RR = 0.93, 95% CI: 0.90–0.96, *p* < 0.001). Disease-specific analyses revealed that in low-SNAP areas, encounters for kidney disease declined modestly (RR = 0.85, 95% CI: 0.71–1.02, *p* = 0.073), whereas in high-SNAP areas, diabetes mellitus showed a marginal reduction (RR = 0.89, 95% CI: 0.79–1.01, *p* = 0.069), and hyperthyroidism exhibited a marginal increase (RR = 1.47, 95% CI: 0.94–2.29, *p* = 0.092).

### Excess healthcare visits attributable to COVID-19 exposure

To estimate the absolute impact of COVID-19 exposure on healthcare utilization, we converted the DiD RRs into excess healthcare visits per 1,000 exposed individuals (Table 3). Negative values indicate fewer visits than expected, while positive values reflect excess utilization attributable to COVID-19 exposure.

**Table 3:** Estimated excess healthcare visits attributable to COVID-19 exposure, expressed as the number of additional or fewer encounters per 1,000 patients. Values represent differences in post- versus pre-index periods based on difference-in-differences models, with 95% confidence intervals. Bold for *p* ≤ 0.10

	All Encounters	Emergency	Inpatient	Outpatient	Virtual
All Encounters	**−262.2** **(−385.7 to −140.8)**	−77.1 (−197.0 to 35.2)	−90.5 (−226.0 to 34.7)	**−227.0** **(−343.1 to −113.0)**	−60.2 (−211.6 to 83.0)
Anxiety Disorders	23.3 (−195.8 to 237.1)	**−202.6** **(−414.7 to −9.7)**	−119.8 (−360.9 to 93.1)	90.7 (−110.0 to 285.8)	−18.5 (−277.1 to 220.9)
Depressive Disorders	**255.0** **(−23.7 to 525.6)**	−117.0 (−361.3 to 102.4)	−124.2 (−387.1 to 105.1)	109.3 (−151.8 to 361.7)	−190.4 (−571.9 to 156.0)
Diabetes Mellitus	**−551.4** **(−767.9 to −340.3)**	−51.9 (−242.1 to 120.3)	−82.6 (−292.6 to 105.0)	**−451.8** **(−657.7 to −251.5)**	−173.1 (−436.5 to 68.5)
Drug Poisoning / Overdose	**−1,393.3** **(−1,817.3 to −984.1)**	**−413.4** **(−726.7 to −134.9)**	−212.1 (−491.0 to 34.1)	**−1,159.9** **(−1,583.5 to −753.4)**	−12.4 (−484.1 to 400.9)
Heart Failure	**−630.2** **(−1,056.2 to −219.9)**	−60.8 (−399.8 to 230.3)	−106.2 (−386.2 to 138.8)	**−519.4** **(−929.6 to −126.4)**	−15.6 (−477.4 to 384.4)
Hyperthyroidism	**1,283.5** **(435.9 to 2,059.0)**	−370.3 (−1,359.4 to 286.6)	536.0 (−155.0 to 986.4)	681.9 (−143.0 to 1,428.3)	71.2 (−950.3 to 848.3)
Hypothyroidism	**−852.5** **(−1,257.8 to −463.5)**	−343.1 (−777.9 to 17.8)	−164.0 (−557.1 to 158.2)	**−641.2** **(−1,018.6 to −280.7)**	215.0 (−167.1 to 548.6)
Kidney Disease	**−1,267.2** **(−1,583.5 to −959.7)**	−203.7 (−459.8 to 24.3)	−150.1 (−362.7 to 40.7)	**−893.8** **(−1,198.1 to −599.1)**	−185.1 (−528.4 to 123.6)
Malignant Neoplasms	50.5 (−362.0 to 447.7)	−31.7 (−370.9 to 249.5)	−30.7 (−396.5 to 273.0)	69.1 (−317.3 to 439.7)	95.0 (−324.0 to 460.9)

Overall, there was a significant reduction of 262 visits per 1,000 individuals (95% CI: −386 to −141), primarily driven by decreases in outpatient visits (−227 visits; 95% CI: −343 to −113). Changes in emergency, inpatient, and virtual visit were not statistically significant at the population level.

When examining subgroup of specific study disease conditions, notable reductions in excess visits were observed for several chronic disease cohorts, including diabetes mellitus (−551 visits; 95% CI: −768 to −340), hypothyroidism (−853 visits; 95% CI: −1,258 to −464), kidney disease (−1,267 visits; 95% CI: −1,584 to −960), and heart failure (−630 visits; 95% CI: −1,056 to −220). These declines were most pronounced in outpatient care, suggesting substantial disruptions in routine management of chronic conditions, for patients with a positive COVID-19 test. Similarly, visits related to drug poisoning/overdose decreased significantly by 1,393 visits per 1,000 (95% CI: −1,817 to −984), particularly in emergency and outpatient settings.

Conversely, hyperthyroidism was associated with a significant excess of 1,284 visits per 1,000 individuals (95% CI: 436 to 2,059), potentially reflecting increased monitoring or follow-up care post-exposure. 

No significant changes were observed for malignant neoplasms or depressive disorders, though wide confidence intervals suggest heterogeneity and possible underpowering.

## Discussion

### Principal findings

In this large, population-based cohort study, we found that exposure to COVID-19 was associated with a significant and heterogeneous decline in healthcare utilization across clinical settings, disease categories, and sociodemographic groups. DiD analyses showed that individuals who tested positive for COVID-19 had 4% lower healthcare utilization in the six months following diagnosis relative to matched controls, with the largest relative reductions seen in outpatient visits. This finding reflects a differential pattern in post-COVID utilization, not an absolute decline, as utilization decreased for both groups compared to the contiguous pre-index period but did not return to the lower levels observed during the baseline pre-index period. The reductions in utilization were particularly pronounced among patients with diabetes mellitus, heart failure, kidney disease, and hypothyroidism—conditions that require consistent, longitudinal management to prevent acute decompensation. Interruptions in routine care may therefore represent missed opportunities for early intervention and ongoing management, potentially leading to long-term adverse outcomes [16, 17]. Utilization related to mental health conditions—including anxiety and depressive disorders—did not significantly differ between COVID-positive individuals and matched controls in the post-index period. This suggests that mental health care demand persisted across groups, potentially supported by rapid adoption of telehealth during the pandemic, aided by regulatory flexibilities and continued need for behavioral health services [13, 14]. Older adults, males, and Black individuals experienced significantly greater *relative* declines in healthcare encounters compared to matched controls. These findings suggest that COVID-19 exposure disproportionately disrupted care among populations already facing structural or behavioral barriers to engagement. For example, prior research has shown that men are less likely to seek medical care than women, due in part to sociocultural norms surrounding masculinity [37, 38]. Among older adults—especially those over age 80—decreased utilization may reflect a preference for self-management or a tendency to attribute symptoms to aging [39]. In the case of Black patients, higher relative reductions in outpatient use may reflect systemic barriers to access and delayed presentation, consistent with prior studies showing elevated hospitalization rates and more severe illness at time of care [40, 41]. Medicare beneficiaries also experienced a decrease in utilization. Patients residing in areas with lower income, lower educational attainment, or higher SNAP enrollment had similarly reduced utilization. These patterns may reflect a range of access barriers, including logistical challenges, limited availability of telehealth infrastructure, and heightened concerns about in-person visits during the pandemic [24, 25]. Such reductions may represent the compounding effects of financial hardship, competing survival demands, and longstanding mistrust in healthcare systems [26, 27].

Disease-specific DiD shows a persistent decline in outpatient encounters were most notable for kidney disease and drug poisoning/overdose. Older adults (≥65) saw an 11% reduction in diabetes visits, while adults aged 35–54 experienced a 22% drop in kidney disease visits. Males showed marked decreases in encounters for both drug poisoning/overdose and kidney disease. Individuals identifying as Multiple/Other/Unknown ethnicity had a striking 55% decline in drug poisoning/overdose visits, highlighting potential structural barriers to care. Unexpectedly, uninsured patients (Self-Pay) showed increased visits for heart failure and hyperthyroidism, suggesting care was sought despite lack of coverage. Reductions in kidney disease visits were also evident among typically well-resourced groups, including Medicare beneficiaries, residents of high-income areas, and individuals with obesity.

#### Public health and policy Implications

Our findings have important implications for post-pandemic recovery and future health system resilience. The observed declines in care for chronic conditions and in marginalized populations suggest the potential for worsening health inequities in the years to come. As this study aimed to isolate the impact of COVID-19 exposure on healthcare utilization, the disproportionate reductions among exposed individuals highlight how the infection itself may have directly disrupted engagement with care. Delayed care may contribute to later-stage disease presentation, excess morbidity, and elevated health system burden—particularly in primary care and emergency departments [28, 29]. Moreover, a one-size-fits-all approach to care restoration may fail to meet the needs of groups disproportionately affected by the pandemic.

Policymakers and health system leaders must prioritize targeted, equity-driven interventions to address these disparities. Strategies may include expanding community-based outreach, strengthening culturally competent care delivery, enhancing digital infrastructure for telehealth in underserved regions, and increasing investments in social determinants of health (e.g., food and housing security) [30, 31]. In particular, the differential telehealth uptake observed across conditions and populations points to its promise as both a bridge during disruption and a long-term tool to mitigate geographic and structural barriers [42, 43].

Finally, the interaction between chronic disease, nutritional insecurity, and pandemic exposure underscores the importance of integrated care models that account for both clinical and social risk. For example, food insecurity has been linked to worse control of diabetes, possibly due to nutritional instability and reduced treatment adherence [44, 45]. Addressing these upstream factors may be essential to promoting equitable recovery and minimizing the long-term consequences of deferred care.

### Limitations

This study has several important limitations. First, our findings derive from data within a single health system, which may limit the generalizability of results to other geographic regions, healthcare settings, or populations with different insurance structures. Second, the retrospective design relies on structured electronic health record (EHR) and administrative data, which are subject to potential inaccuracies, incomplete documentation, and misclassification—particularly regarding insurance type and the “Other/Unknown” categories. Third, patients with multiple chronic conditions may appear in several condition-specific cohorts, potentially confounding the independent effect of each condition on healthcare utilization patterns. Additionally, because some COVID-19–positive patients had multiple study diseases, the exposure cohort may have a higher overall disease burden than the control cohort. Fourth, socioeconomic and demographic variables were categorized using percentile-based cutoffs to enhance interpretability, but this approach may introduce classification bias depending on the underlying data distribution. Fifth, the study lacks detailed clinical severity measures beyond obesity, limiting our ability to adjust for disease severity—a factor influencing healthcare utilization, this is however somewhat mitigated by the case-control design. This could influence healthcare utilization independently of COVID-19 exposure and should be considered when interpreting the results. Future research incorporating more granular use of clinical data would enable more precise risk adjustment and interpretation. Sixth, although a six-month post-exposure follow-up period is consistent with prior studies, it may not fully capture longer-term shifts in care-seeking behavior or adaptations within the health care system. More granular and extended temporal analyses could offer deeper insights into evolving trends in health care utilization and outcomes over time. Seventh, given the exploratory nature of our analyses across multiple conditions and subgroups, we did not apply formal corrections for multiple comparisons. As such, findings should be interpreted with caution, and future studies may consider more stringent statistical controls to confirm observed patterns. Finally, we did not account for patients’ COVID-19 vaccination status, which can influence both exposure risk and subsequent health care utilization. However, our study period—from March 11, 2020, to December 17, 2020—largely predates the availability of COVID-19 vaccines. The first vaccine (Pfizer-BioNTech) received Emergency Use Authorization in the U.S. on December 11, 2020, with distribution starting shortly afterward. Therefore, vaccination status is unlikely to have significantly impacted our findings during this timeframe. Nonetheless, the lack of vaccination data limits our ability to fully interpret longer-term changes in health care engagement occurring in later phases of the pandemic.

## Conclusions

Our study reveals significant and heterogeneous changes in healthcare utilization following COVID-19 exposure, with marked declines in outpatient visits and chronic disease management, and notable disparities across demographic, socioeconomic, and insurance subgroups. The observed increases in virtual care utilization highlight the rapid adaptation of healthcare delivery during the pandemic, yet persistent heterogenous care utilization underscore ongoing challenges in equitable access.

These findings have important implications for health system planning and policy development. The enduring impact of the COVID-19 pandemic on healthcare systems and patient well-being is likely to persist for years, demanding sustained efforts to monitor, understand, and mitigate disruptions in care. Of particular concern are the potential long-term consequences for individuals with chronic diseases, mental health conditions, and substance use disorders, populations already vulnerable to healthcare disparities exacerbated by socio-economic contextual factors.

By elucidating the differential impact of the pandemic on healthcare utilization across diverse patient groups, this study informs public health strategies aimed at reducing inequities. The expanded use of telemedicine and other innovative care models may provide critical avenues to enhance access and continuity of care, particularly for underserved populations.

Moreover, our use of high-granularity, population-level data—stratified by clinical condition, demographics, and socioeconomic context—demonstrates the value of disease- and context-specific analytics which could serve as a foundation for future situational awareness and predictive analytics during ongoing or emerging public health crises [46].

Ultimately, our findings highlighting the need for targeted policies to ensure care continuity and equity to mitigate management disruption health system adapts to ongoing challenges during a pandemic, sustained efforts are needed to address structural inequities and re-engage patients in essential care.

## Electronic supplementary material

Below is the link to the electronic supplementary material.

Supplementary Material 1

## Data Availability

The data sets generated for and analyzed during this study are not publicly available due the approved study protocol restrictions and patient privacy considerations. The data can be made available following a study protocol review and an appropriate institutional review board approval.

## Abbreviations

EHR: Electronic Health Records
ACS: American Community Survey
SNAP: Supplemental Nutrition Assistance Program
ICD-9/10: International Classification of Diseases, Ninth/Tenth Revision
DID: Difference-In-Difference
COVID-19: Novel-COronaVIrus Disease 2019
RR: Rate Ratio

## Footnotes

1 The dataset includes patient demographic details, including self-reported sex, age, race, and ethnicity when available, as well as residential zip code and deceased status. In cases where self-reporting is unavailable, demographic information may be recorded by hospital staff based on existing records. The dataset also includes individual-level healthcare utilization, such as inpatient admissions, emergency room visits, and outpatient (clinic-based and telehealth) appointments. These encounters include information on the location of care, reason for the visit, and diagnosis.
