# Supplementary material for: Health care utilization before and after COVID-19 diagnosis: a multidisease matched-cohort study by sociodemographic factors
Source: BMC Health Serv Res. 2026 Jun 24;26:1021. doi: 10.1186/s12913-026-14934-z (PMC13401288; doi:10.1186/s12913-026-14934-z)
Supplement: Supplementary file 1 — Supplementary Material 1 [file 12913_2026_14934_MOESM1_ESM.pdf]

Supplementary material for

Health Care Utilization Before and After COVID-19 Diagnosis: A Multidisease Matched-Cohort Study by Sociodemographic Factors

Alina Peluso[1], Omar Aljawfi[2], Kelley M. Anderson[3], Margret V. Bjarnadottir[4], Nawar Shara[2]

[1] Oak Ridge National Laboratory, Oak Ridge, TN, 37830, United States
[2] MedStar Health Research Institute, Hyattsville, MD, 21044, United States
[3] University of Virginia, Charlottesville, VA, 22903, United States
[4] University of Maryland, College Park, MD, 20742, United States

Appendix A

To estimate the impact of COVID-19 infection on healthcare utilization, we employed a difference-in-differences (DiD) analytical framework implemented via Poisson regression. Poisson models were selected due to their suitability for count data and common use in health services research to evaluate encounter rates. The primary outcome was healthcare utilization, defined as the number of unique clinical encounters per patient.

We compared encounter rates over two six-month observation periods: (1) a post-index period, beginning 14 days after the COVID-19 test result (positive or negative), and (2) a baseline pre-index period, defined as the same calendar months one year prior. This design accounts for seasonality and enables isolation of the effect of COVID-19 infection from concurrent secular trends.
To aid interpretation, adjusted rate ratios were translated into absolute differences in encounters per 1,000 COVID-19 positive patients using observed visit volumes in the post-period.

Poisson DiD Model Specification

The model is of the form:

$$E[Y_{it}] = \exp(\beta_0 + \beta_1 \cdot post_t + \beta_2 \cdot case_i + \beta_3 \cdot (post_t \times case_i)$$

where:

- Y_{it} is the number of encounters for individual *i* in time period *t*
- $post_t$ is a binary indicator for the post-index period
- $post_t \times case_i$ is the interaction term capturing the DiD effect.

This model estimates the log of the expected encounter count, and the coefficients are interpreted as log-rate effects. Specifically, the coefficient β_3 on the interaction term captures the differential change in encounter rates for the exposed group after the index period, compared to the control group. Additionally, because the case indicator (β_2) captures all time-invariant individual characteristics, when pre-to-post differences are computed, the effects of these stable factors cancel out, so the DiD coefficient (β_3) is not biased by baseline health status or other constant individual-level confounders.

Exponentiating the coefficients gives the multiplicative effect on the expected rate. Thus, the term:

$$\text{Rate Ratio (RR)} = \exp(\beta_3) = \frac{\text{(post/pre rate in controls)}}{\text{(post/pre rate in cases)}}$$

Which is interpreted as the **relative change in the encounter rate** for the exposed group in the post-index period, compared to the unexposed group, adjusting for baseline and time effects.

Excess Visits per 1,000 Patients

To translate the relative effect into an absolute burden, we estimated the number of **excess encounters per 1,000 individuals** attributable to the exposure using:

$$\text{Excess Visits per 1,000} = (N\_case\_post * (1 - 1 / RR)) * (1000 / n\_case\_post)$$

where:

- RR is the rate ratio from the interaction term
- N_case_post is the total number of encounters observed in the exposed group post-index
- n_case_post is the number of individuals in the exposed group post-index

This approach provides an interpretable estimate of the number of encounters attributable to the exposure, scaled per 1,000 individuals, while adjusting for time trends observed in controls.

Supplementary Figures and Tables

Supplementary Table 1. Diagnosis code used to select the patients in the respective cohorts.

Disease	*Description*	*ICD-10 Codes*	*ICD-9 Codes*
**Anxiety Disorders**	Anxiety, stress-related, somatoform disorders	F40–F48	300.2, 300.01, 300.02, 300.3, 308.x, 309.81, 300.11, 300.7, 300.5, 300.6
**Depressive Disorders**	Major depressive episodes	F32.x, F33.x	296.2–296.5, 311
**Drug Poisoning / Overdose**	Poisoning by drugs	T36–T50	960–979
**Diabetes Mellitus**	All types of diabetes	E08–E11, E13, E09	250.0–250.9
**Heart Failure**	Congestive/unspecified heart failure	I50.x	428.0–428.9
**Hyperthyroidism**	Overactive thyroid	E05.x	242.x
**Hypothyroidism**	Underactive/iatrogenic thyroid function	E02–E03, E890	243–244
**Kidney Disease**	Acute or chronic kidney disease	N17.x, N18.x	584.x, 585
**Malignant Neoplasms**	General cancers	C00–C97	140–239
**COVID-19 Positive**	Confirmed or history of COVID-19	U07.%, U09.%	–
**COVID-19 Negative / Screening**	Screening/absence/contact exposure	Z03.81.%, Z11.59.%, Z20.82.%	–

Supplementary Table 2. Documentation of the socioeconomic indicators at the U.S. Census tract level in Washington, D.C., Maryland, and Virginia, sourced from the American Community Survey (ACS) for the year 2020.

Domain / Variable Name	*Description*	*Relevance to Healthcare Utilization*
**Income**<br>ACS_MEDIAN_HH_INC	Median household income<br>(dollars, inflation-adjusted to data file year)	Income levels affect affordability of care, insurance coverage, and medication adherence.
**Education**<br>ACS_PCT_GRADUATE_DGR	Percentage of population with a master's, professional school degree, or doctorate (ages 25 and over)	Educational attainment is linked to health literacy, care-seeking behavior, and chronic disease outcomes.
**Poverty/Food Security**<br>ACS_PCT_HH_FOOD_STMP_BLW_POV	Percentage of households receiving food stamps/SNAP benefits with income below the poverty level	Reflects food insecurity, which is linked to chronic disease risk and poor care management.

Supplementary Table 3. Patient counts by condition and COVID-19 status before matching. Conditions are not mutually exclusive — patients may appear in more than one row if they had multiple conditions.

	COVID-19 Negative	*COVID-19 Positive*
**Total Patients**	47,542	10,651
**Anxiety Disorders**	21,876	4,414
**Malignant Neoplasms**	9,646	1,447
**Depressive Disorders**	14,130	2,786
**Diabetes Mellitus**	16,926	4,923
**Drug Poisoning / Overdose**	6,524	1,732
**Heart Failure**	6,473	1,529
**Hyperthyroidism**	1,412	270
**Hypothyroidism**	8,127	1,534
**Kidney Disease**	10,624	2,939

Counts represent individuals in the final geocoded analytic cohort with valid census tract data from Washington, DC; Maryland (MD); or Virginia (VA), who were successfully linked to diagnosis records and stratified by COVID-19 test status. For each COVID-19 positive patient, only one COVID-19 negative patient was selected for subsequent analysis. Patients may serve as a case for more than one condition, and similarly, controls may be included across multiple disease categories. The total number of distinct patients included in the study across all conditions and the disease-specific patient counts are provided in ***Table 1****.*

Supplementary Table 4. Count of encounters for matched patients by disease, period, encounter type and insurance status.

	Pre-Index Period (1 year prior)	Pre-Index Period (Contiguous)	Post-Index Period	Pre-Index Period (1 year prior)	Pre-Index Period (Contiguous)	Post-Index Period
	Case / Control	Case / Control	Case / Control	Case / Control	Case / Control	Case / Control
		**Anxiety Disorders**			**Depressive Disorders**	
**Total**	19,033 / 21,284	32,324 / 34,944	29,398 / 32,072	14,092 / 15,233	21,944 / 24,180	20,058 / 22,029
**Encounter Type**						
… Emergency	1,493 (7.8%) / 1,642 (7.7%)	2,749 (8.5%) / 2,474 (7.1%)	1,672 (5.7%) / 1,844 (5.8%)	1,145 (8.1%) / 1,343 (8.8%)	1,839 (8.4%) / 1,826 (7.6%)	1,227 (6.1%) / 1,424 (6.5%)
… Inpatient	815 (4.3%) / 894 (4.2%)	2,494 (7.7%) / 1,853 (5.3%)	996 (3.4%) / 1,148 (3.6%)	693 (4.9%) / 717 (4.7%)	2,023 (9.2%) / 1,514 (6.3%)	845 (4.2%) / 942 (4.3%)
… Outpatient	15,040 (79%) / 17,062 (80.2%)	21,920 (67.8%) / 25,637 (73.4%)	21,582 (73.4%) / 23,946 (74.7%)	11,172 (79.3%) / 12,158 (79.8%)	14,806 (67.5%) / 17,551 (72.6%)	14,522 (72.4%) / 16,288 (73.9%)
… Virtual	1,685 (8.8%) / 1,686 (7.9%)	5,161 (16%) / 4,980 (14.2%)	5,148 (17.5%) / 5,134 (16%)	1,082 (7.7%) / 1,015 (6.7%)	3,276 (14.9%) / 3,289 (13.6%)	3,464 (17.3%) / 3,375 (15.3%)
**Insurance**						
… Commercial	5,507 (28.9%) / 6,288 (29.5%)	8,921 (27.6%) / 9,715 (27.8%)	8,216 (28%) / 8,784 (27.4%)	3,606 (25.6%) / 4,112 (27%)	5,377 (24.5%) / 6,066 (25.1%)	4,972 (24.8%) / 5,244 (23.8%)
… Medicare	4,852 (25.5%) / 5,600 (26.3%)	7,041 (21.8%) / 8,079 (23.1%)	6,398 (21.8%) / 7,820 (24.4%)	4,116 (29.2%) / 4,892 (32.1%)	5,871 (26.8%) / 7,055 (29.2%)	5,156 (25.7%) / 6,574 (29.8%)
… Self Pay	136 (0.7%) / 150 (0.7%)	342 (1.1%) / 264 (0.8%)	236 (0.8%) / 185 (0.6%)	100 (0.7%) / 86 (0.6%)	170 (0.8%) / 159 (0.7%)	90 (0.4%) / 111 (0.5%)
… Veteran/Amerigroup/Other Government	604 (3.2%) / 747 (3.5%)	937 (2.9%) / 1,161 (3.3%)	805 (2.7%) / 1,010 (3.1%)	495 (3.5%) / 440 (2.9%)	707 (3.2%) / 703 (2.9%)	625 (3.1%) / 612 (2.8%)
… Other/Unknown	7,934 (41.7%) / 8,499 (39.9%)	15,083 (46.7%) / 15,725 (45%)	13,743 (46.8%) / 14,273 (44.5%)	5,775 (41%) / 5,703 (37.4%)	9,819 (44.8%) / 10,197 (42.2%)	9,215 (45.9%) / 9,488 (43.1%)
		**Drug Poisoning / Overdose**			**Heart Failure**	
**Total**	8,788 / 10,815	13,062 / 17,126	12,742 / 17,648	8,284 / 9,502	12,085 / 14,695	11,765 / 14,410
**Encounter Type**						
… Emergency	942 (10.7%) / 1,052 (9.7%)	1,341 (10.3%) / 1,682 (9.8%)	974 (7.6%) / 1,391 (7.9%)	606 (7.3%) / 606 (6.4%)	919 (7.6%) / 900 (6.1%)	647 (5.5%) / 694 (4.8%)
… Inpatient	740 (8.4%) / 929 (8.6%)	2,247 (17.2%) / 1,896 (11.1%)	961 (7.5%) / 1,299 (7.4%)	712 (8.6%) / 764 (8%)	2,090 (17.3%) / 1,669 (11.4%)	863 (7.3%) / 1,003 (7%)
… Outpatient	6,584 (74.9%) / 8,214 (76%)	8,160 (62.5%) / 11,785 (68.8%)	9,195 (72.2%) / 13,027 (73.8%)	6,513 (78.6%) / 7,602 (80%)	8,017 (66.3%) / 10,655 (72.5%)	8,915 (75.8%) / 11,174 (77.5%)
… Virtual	522 (5.9%) / 620 (5.7%)	1,314 (10.1%) / 1,763 (10.3%)	1,612 (12.7%) / 1,931 (10.9%)	453 (5.5%) / 530 (5.6%)	1,059 (8.8%) / 1,471 (10%)	1,340 (11.4%) / 1,539 (10.7%)
**Insurance**						
… Commercial	1,682 (19.1%) / 2,660 (24.6%)	2,451 (18.8%) / 3,897 (22.8%)	2,504 (19.6%) / 4,142 (23.5%)	1,735 (20.9%) / 1,946 (20.5%)	2,137 (17.7%) / 3,100 (21.1%)	2,201 (18.7%) / 3,067 (21.3%)
… Medicare	3,456 (39.3%) / 4,483 (41.5%)	4,917 (37.6%) / 6,614 (38.6%)	4,634 (36.4%) / 6,783 (38.4%)	3,937 (47.5%) / 4,809 (50.6%)	5,696 (47.1%) / 7,069 (48.1%)	5,298 (45%) / 6,709 (46.6%)
… Self Pay	65 (0.7%) / 91 (0.8%)	112 (0.9%) / 123 (0.7%)	64 (0.5%) / 68 (0.4%)	40 (0.5%) / 43 (0.4%)	103 (0.8%) / 72 (0.5%)	98 (0.8%) / 35 (0.2%)
… Veteran/Amerigroup/Other Government	271 (3.1%) / 260 (2.4%)	382 (2.9%) / 346 (2%)	343 (2.7%) / 372 (2.1%)	145 (1.8%) / 129 (1.4%)	156 (1.3%) / 207 (1.4%)	161 (1.4%) / 207 (1.4%)
… Other/Unknown	3,314 (37.7%) / 3,321 (30.7%)	5,200 (39.8%) / 6,146 (35.9%)	5,197 (40.8%) / 6,283 (35.6%)	2,427 (29.3%) / 2,575 (27.1%)	3,993 (33%) / 4,247 (28.9%)	4,007 (34.1%) / 4,392 (30.5%)
		**Hypothyroidism**			**Kidney Disease**	
**Total**	7,193 / 7,683	11,299 / 12,922	10,038 / 11,836	14,277 / 17,260	21,415 / 27,500	19,356 / 27,385
**Encounter Type**						
… Emergency	391 (5.4%) / 376 (4.9%)	729 (6.4%) / 592 (4.6%)	466 (4.6%) / 483 (4.1%)	967 (6.8%) / 1,106 (6.4%)	1,570 (7.3%) / 1,649 (6%)	1,006 (5.2%) / 1,349 (4.9%)
… Inpatient	319 (4.4%) / 350 (4.6%)	1,026 (9.1%) / 729 (5.6%)	366 (3.6%) / 483 (4.1%)	961 (6.7%) / 1,247 (7.2%)	3,617 (16.9%) / 2,944 (10.7%)	1,273 (6.6%) / 1,702 (6.2%)
… Outpatient	5,954 (82.8%) / 6,357 (82.7%)	8,024 (71%) / 9,871 (76.4%)	7,830 (78%) / 9,270 (78.3%)	11,545 (80.9%) / 13,929 (80.7%)	14,241 (66.5%) / 20,143 (73.2%)	14,761 (76.3%) / 21,319 (77.8%)
… Virtual	529 (7.3%) / 600 (7.8%)	1,520 (13.4%) / 1,730 (13.4%)	1,376 (13.7%) / 1,600 (13.5%)	804 (5.6%) / 978 (5.7%)	1,987 (9.3%) / 2,764 (10.1%)	2,316 (12%) / 3,015 (11%)
**Insurance**						
… Commercial	1,915 (26.6%) / 2,099 (27.3%)	2,675 (23.7%) / 3,483 (27%)	2,466 (24.6%) / 3,035 (25.6%)	3,172 (22.2%) / 3,863 (22.4%)	4,290 (20%) / 6,315 (23%)	4,162 (21.5%) / 6,226 (22.7%)
… Medicare	2,770 (38.5%) / 3,300 (43%)	4,053 (35.9%) / 5,039 (39%)	3,495 (34.8%) / 4,654 (39.3%)	6,536 (45.8%) / 8,299 (48.1%)	9,370 (43.8%) / 12,695 (46.2%)	8,035 (41.5%) / 12,344 (45.1%)
… Self Pay	33 (0.5%) / 25 (0.3%)	99 (0.9%) / 37 (0.3%)	72 (0.7%) / 35 (0.3%)	89 (0.6%) / 80 (0.5%)	139 (0.7%) / 92 (0.3%)	127 (0.7%) / 64 (0.2%)
… Veteran/Amerigroup/Other Government	130 (1.8%) / 125 (1.6%)	200 (1.8%) / 239 (1.9%)	177 (1.8%) / 286 (2.4%)	239 (1.7%) / 250 (1.4%)	288 (1.3%) / 404 (1.5%)	269 (1.4%) / 430 (1.6%)
… Other/Unknown	2,345 (32.6%) / 2,134 (27.8%)	4,272 (37.8%) / 4,124 (31.9%)	3,828 (38.1%) / 3,826 (32.3%)	4,241 (29.7%) / 4,768 (27.6%)	7,328 (34.2%) / 7,994 (29.1%)	6,763 (34.9%) / 8,321 (30.4%)
		**Diabetes Mellitus**			**Hyperthyroidism**	
**Total**	18,540 / 23,273	29,795 / 38,734	25,789 / 35,435	1,435 / 1,839	2,276 / 2,615	2,131 / 2,440
**Encounter Type**						
… Emergency	1,358 (7.3%) / 1,634 (7%)	2,661 (8.9%) / 2,402 (6.2%)	1,350 (5.2%) / 1,705 (4.8%)	68 (4.7%) / 115 (6.2%)	150 (6.6%) / 131 (5%)	74 (3.5%) / 109 (4.5%)
… Inpatient	913 (4.9%) / 1,111 (4.8%)	3,889 (13.1%) / 2,684 (6.9%)	1,206 (4.7%) / 1,588 (4.5%)	60 (4.2%) / 111 (6%)	186 (8.2%) / 149 (5.7%)	72 (3.4%) / 104 (4.3%)
… Outpatient	15,044 (81.1%) / 19,049 (81.8%)	19,700 (66.1%) / 29,345 (75.8%)	19,726 (76.5%) / 27,814 (78.5%)	1,159 (80.8%) / 1,473 (80.1%)	1,547 (68%) / 2,025 (77.4%)	1,630 (76.5%) / 1,900 (77.9%)
… Virtual	1,225 (6.6%) / 1,479 (6.4%)	3,545 (11.9%) / 4,303 (11.1%)	3,507 (13.6%) / 4,328 (12.2%)	148 (10.3%) / 140 (7.6%)	393 (17.3%) / 310 (11.8%)	355 (16.7%) / 327 (13.4%)
**Insurance**						
… Commercial	4,756 (25.6%) / 6,120 (26.3%)	6,906 (23.2%) / 10,006 (25.8%)	6,020 (23.3%) / 9,032 (25.5%)	387 (27%) / 439 (23.9%)	624 (27.4%) / 657 (25.1%)	603 (28.3%) / 645 (26.4%)
… Medicare	6,862 (37%) / 9,078 (39%)	10,044 (33.7%) / 14,024 (36.2%)	8,593 (33.3%) / 12,784 (36.1%)	524 (36.5%) / 807 (43.9%)	706 (31%) / 990 (37.9%)	671 (31.5%) / 956 (39.2%)
… Self Pay	145 (0.8%) / 123 (0.5%)	355 (1.2%) / 242 (0.6%)	258 (1%) / 195 (0.6%)	3 (0.2%) / 18 (1%)	11 (0.5%) / 15 (0.6%)	2 (0.1%) / 1 (0%)
… Veteran/Amerigroup/Other Government	405 (2.2%) / 527 (2.3%)	639 (2.1%) / 930 (2.4%)	574 (2.2%) / 746 (2.1%)	57 (4%) / 57 (3.1%)	45 (2%) / 58 (2.2%)	49 (2.3%) / 57 (2.3%)
… Other/Unknown	6,372 (34.4%) / 7,425 (31.9%)	11,851 (39.8%) / 13,532 (34.9%)	10,344 (40.1%) / 12,678 (35.8%)	464 (32.3%) / 518 (28.2%)	890 (39.1%) / 895 (34.2%)	806 (37.8%) / 781 (32%)
		**Malignant Neoplasms**				
**Total**	8,032 / 9,205	12,465 / 15,242	12,059 / 14,373			
**Encounter Type**						
… Emergency	403 (5%) / 439 (4.8%)	740 (5.9%) / 604 (4%)	397 (3.3%) / 435 (3%)			
… Inpatient	342 (4.3%) / 376 (4.1%)	1,140 (9.2%) / 801 (5.3%)	478 (4%) / 567 (3.9%)			
… Outpatient	6,728 (83.8%) / 7,851 (85.3%)	9,113 (73.1%) / 12,149 (79.7%)	9,605 (79.7%) / 11,765 (81.8%)			
… Virtual	559 (7%) / 539 (5.9%)	1,472 (11.8%) / 1,688 (11.1%)	1,579 (13.1%) / 1,606 (11.2%)			
**Insurance**						
… Commercial	1,968 (24.5%) / 2,880 (31.3%)	3,125 (25.1%) / 4,521 (29.7%)	3,089 (25.6%) / 4,286 (29.8%)			
… Medicare	3,525 (43.9%) / 3,875 (42.1%)	4,992 (40%) / 5,725 (37.6%)	4,603 (38.2%) / 5,599 (39%)			
… Self Pay	45 (0.6%) / 57 (0.6%)	117 (0.9%) / 87 (0.6%)	106 (0.9%) / 51 (0.3%)			
… Veteran/Amerigroup/Other Government	132 (1.6%) / 181 (2%)	175 (1.4%) / 201 (1.3%)	136 (1.1%) / 206 (1.4%)			
… Other/Unknown	2,362 (29.4%) / 2,212 (24%)	4,056 (32.5%) / 4,708 (30.9%)	4,125 (34.2%) / 4,231 (29.4%)			

Supplementary Figure 1. Geographical distribution of the socioeconomic indicators at the U.S. Census tract level in Washington, D.C., Maryland, and Virginia for the year 2020. Data reflect variation (high, medium, low) across tracts in median household income, educational attainment (percentage with graduate or professional degrees), and the percentage of households receiving food stamps/SNAP below the poverty level, based on the American Community Survey (ACS).

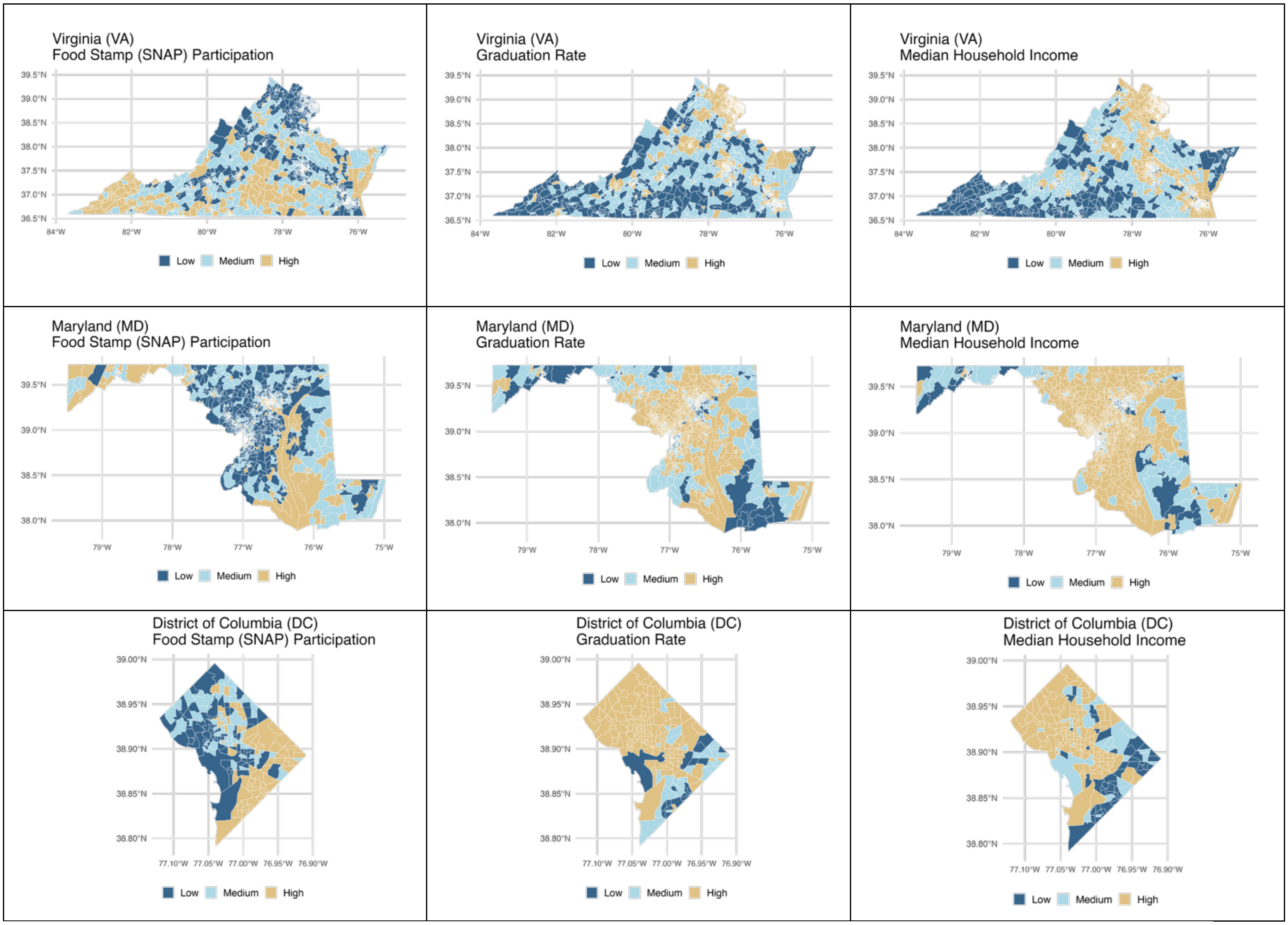

Supplementary Figure 2. Secular trend in matched case-control cohorts by pre-existing condition.

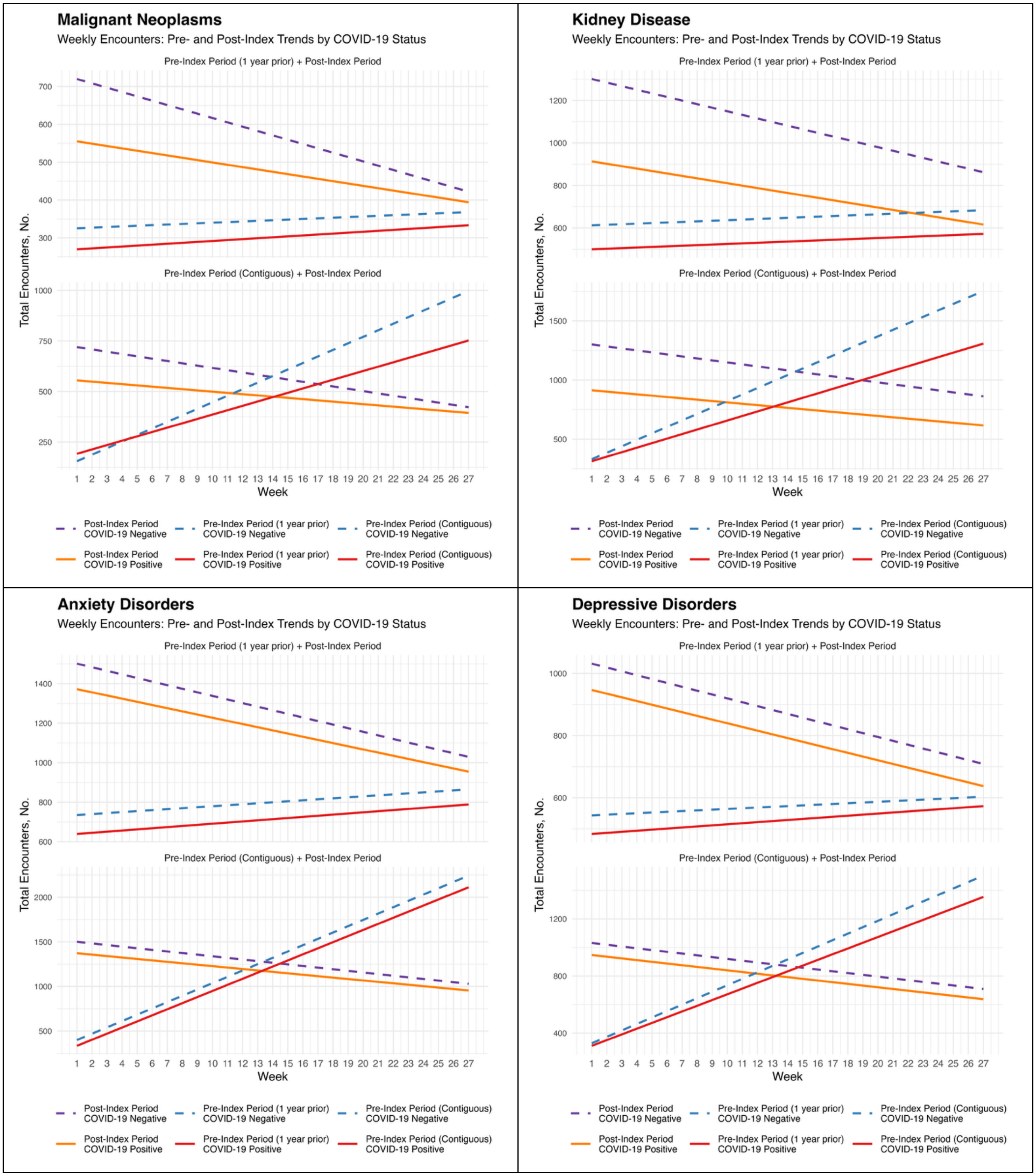

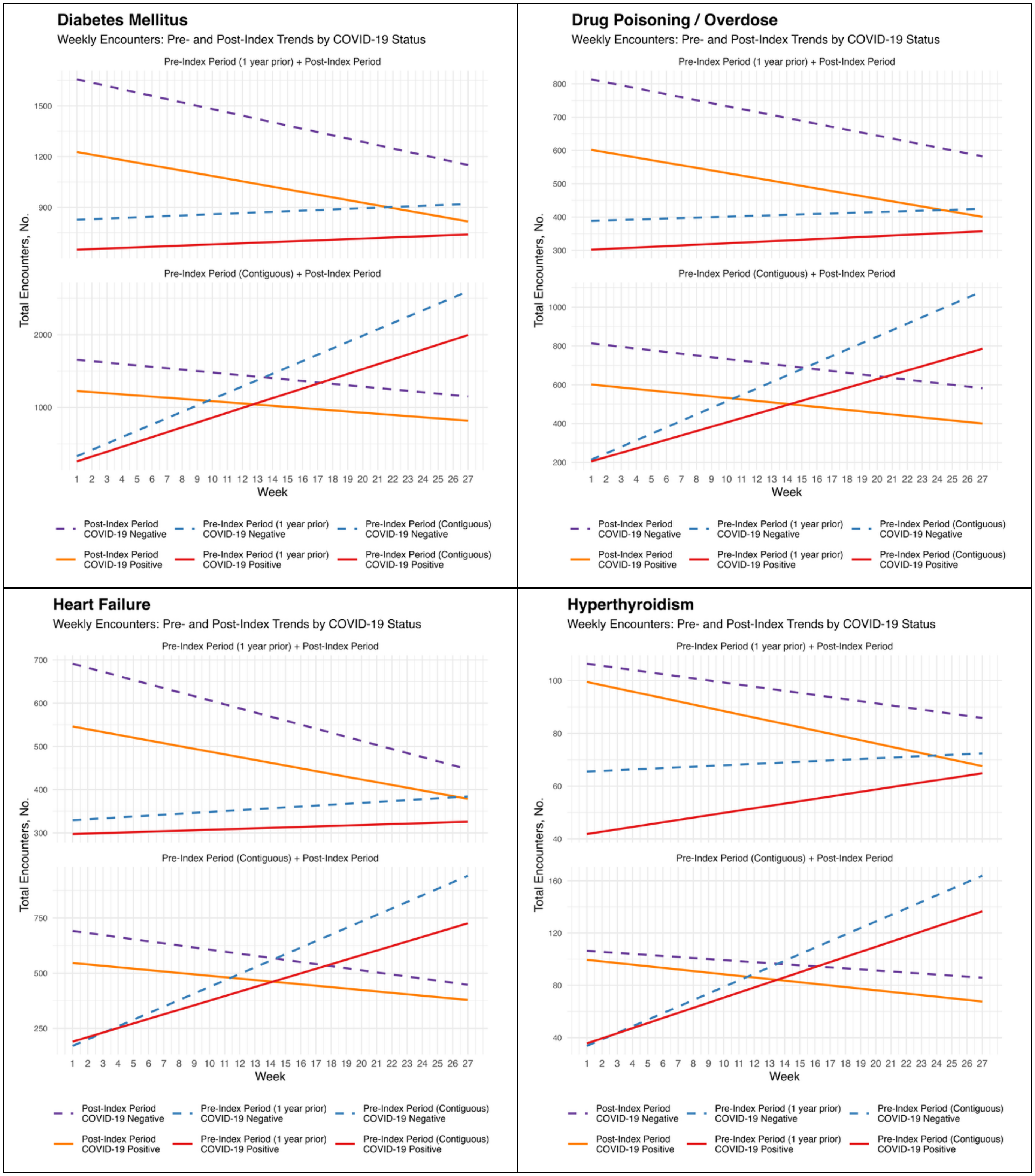

Diabetes Mellitus
Weekly Encounters: Pre- and Post-Index Trends by COVID-19 Status
Pre-Index Period (1 year prior) + Post-Index Period
Pre-Index Period (Contiguous) + Post-Index Period
Total Encounters, No.
Week
Drug Poisoning / Overdose
Weekly Encounters: Pre- and Post-Index Trends by COVID-19 Status
Pre-Index Period (1 year prior) + Post-Index Period
Pre-Index Period (Contiguous) + Post-Index Period
Total Encounters, No.
Week
Heart Failure
Weekly Encounters: Pre- and Post-Index Trends by COVID-19 Status
Pre-Index Period (1 year prior) + Post-Index Period
Pre-Index Period (Contiguous) + Post-Index Period
Total Encounters, No.
Week
Hyperthyroidism
Weekly Encounters: Pre- and Post-Index Trends by COVID-19 Status
Pre-Index Period (1 year prior) + Post-Index Period
Pre-Index Period (Contiguous) + Post-Index Period
Total Encounters, No.
Week
Post-Index Period COVID-19 Negative
Pre-Index Period (1 year prior) COVID-19 Negative
Pre-Index Period (Contiguous) COVID-19 Negative
Post-Index Period COVID-19 Positive
Pre-Index Period (1 year prior) COVID-19 Positive
Pre-Index Period (Contiguous) COVID-19 Positive

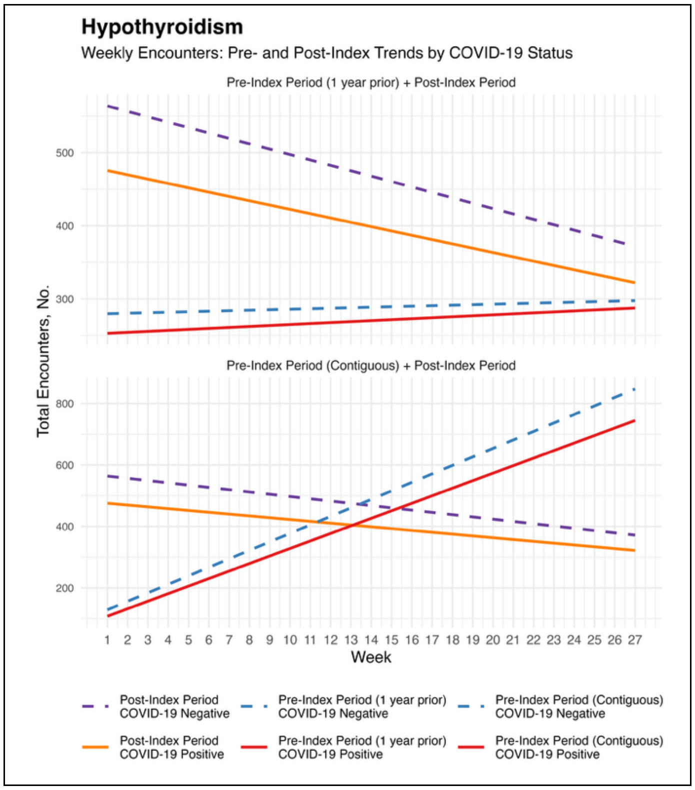

Supplementary Figure 3. Secular trend in matched case-control cohorts by pre-existing condition and encounter type.

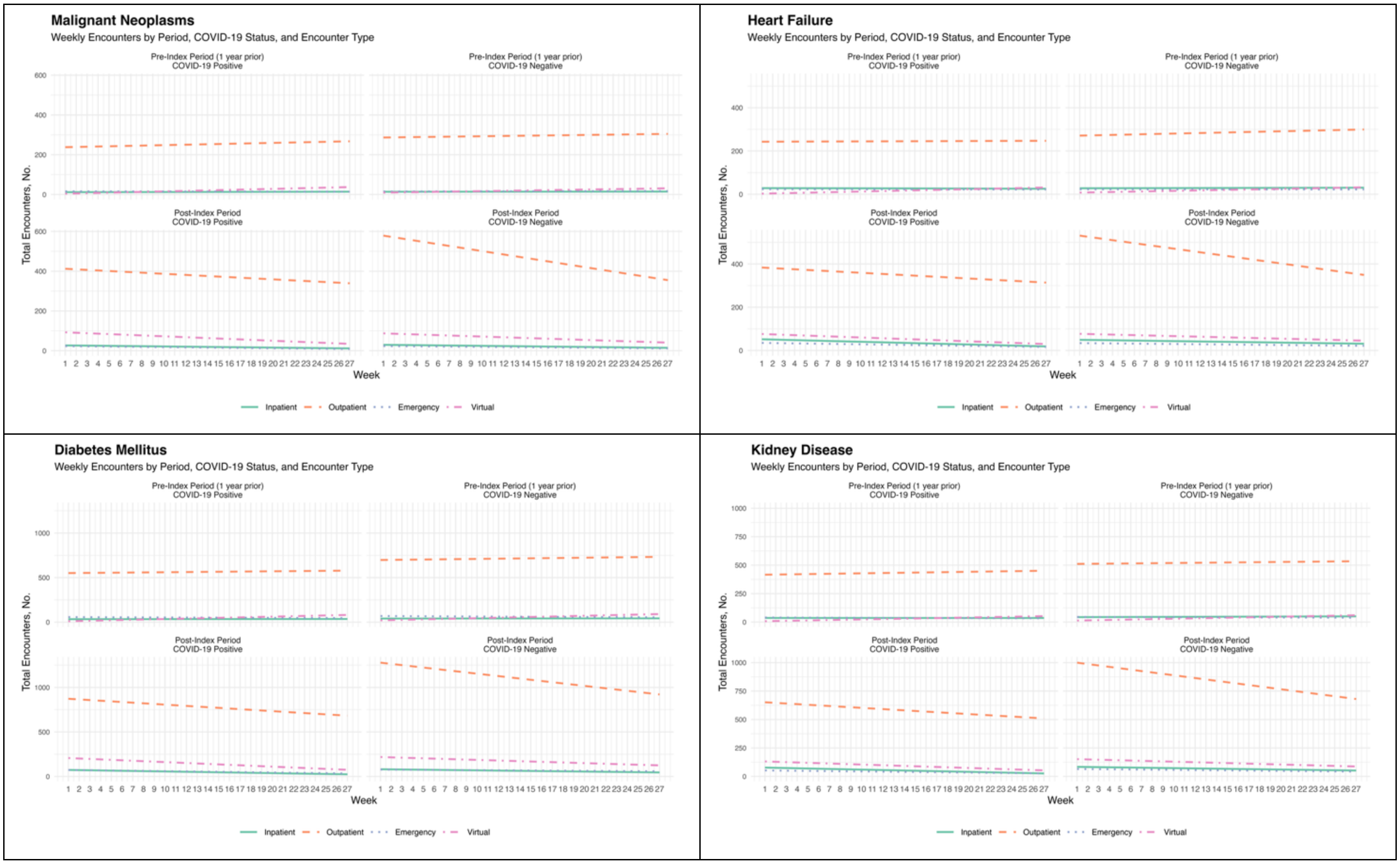

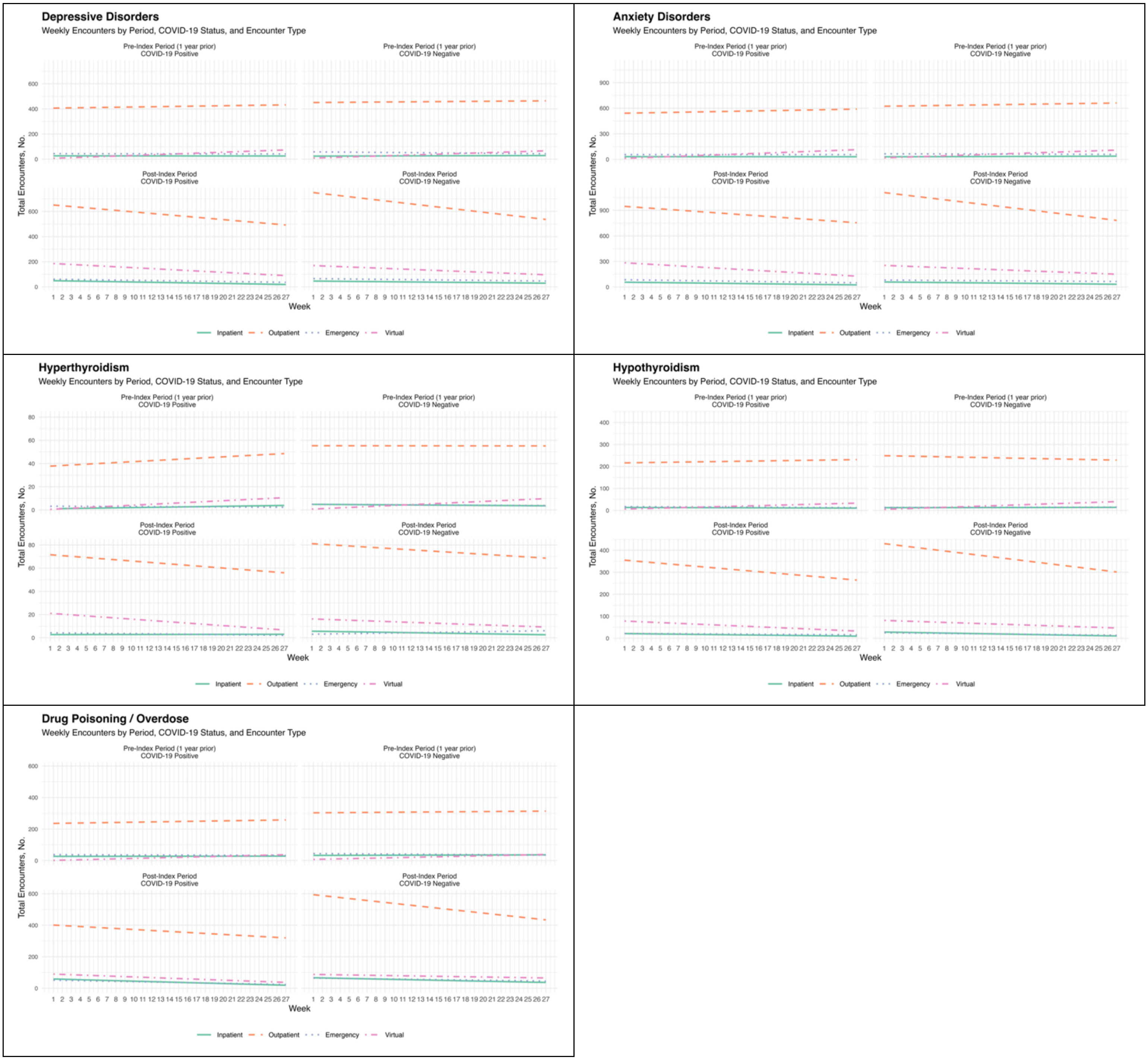

Encounter categories were derived by combining structured information from encounter type and admission type fields. Inpatient *includes inpatient and observation stays.* Outpatient *includes visits classified as outpatient, home health, or preadmission.* Emergency *encounters include only those explicitly labeled as emergency encounters with a corresponding emergency admission.* Virtual *encounters include telehealth visits and secure inbox messages. Encounters with incomplete or conflicting data were excluded.*

Supplementary Figure 4. Healthcare utilization associated with positive versus negative COVID-19 test results. The difference-in-differences parameter represents the adjusted change in encounter rates comparing post- versus pre-periods, estimated using Poisson regression models. Significance codes: "***" p ≤ 0.001; "**" p ≤ 0.01; "*" p ≤ 0.05; "." p ≤ 0.10. Colors indicate disease categories only for statistically significant estimates in bold (p ≤ 0.10).

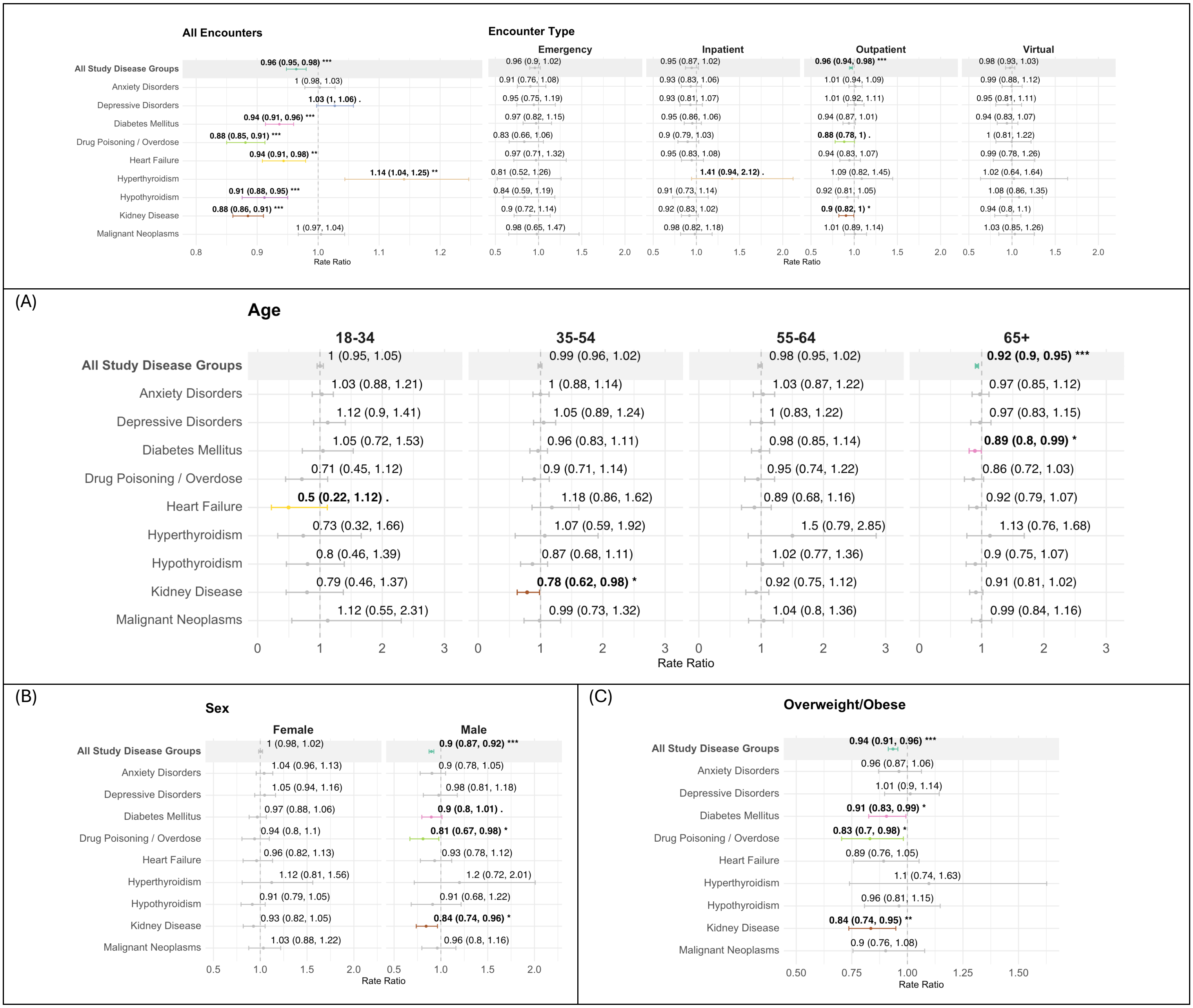

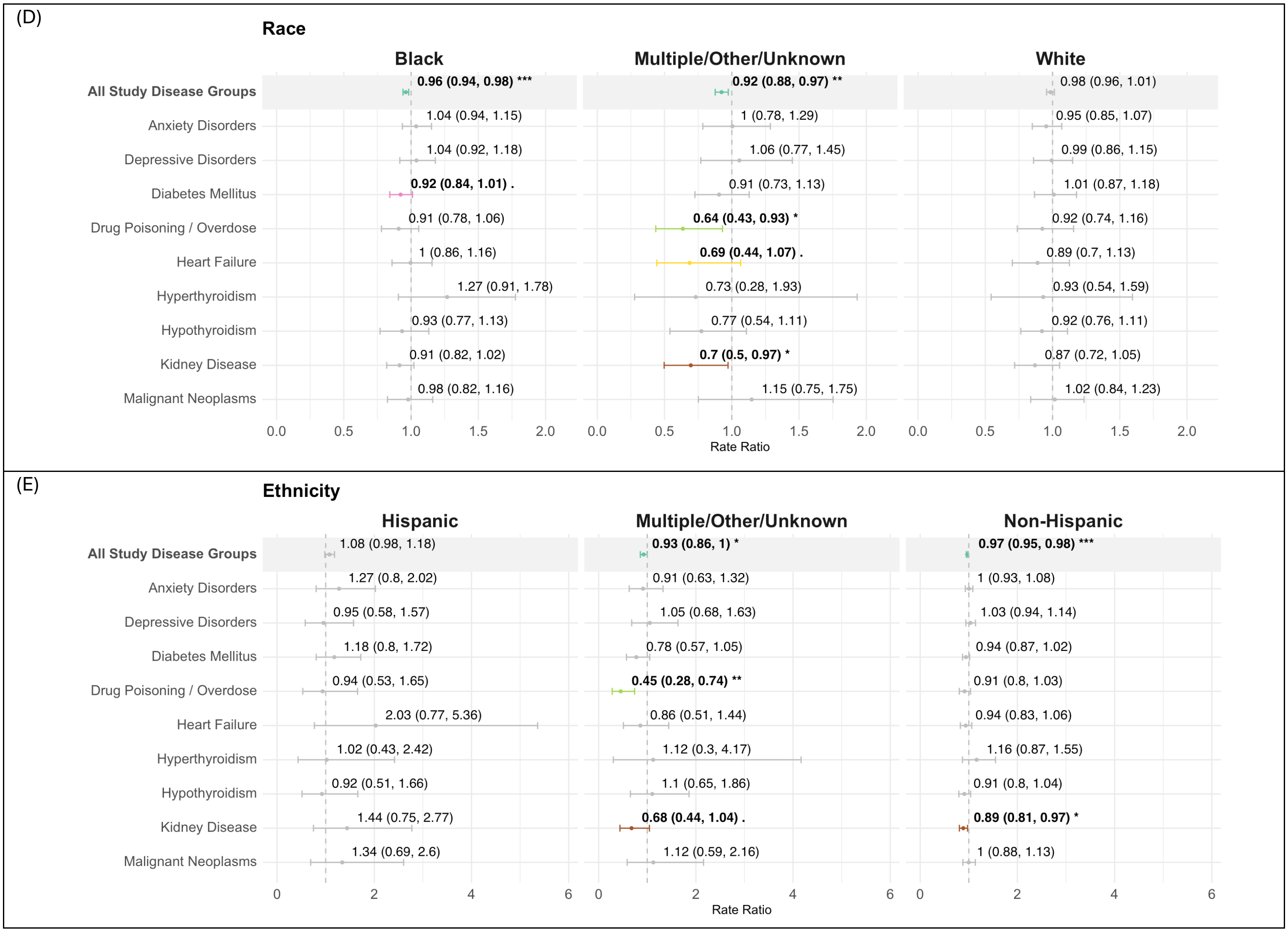
(D)
Race
Black
Multiple/Other/Unknown
White
All Study Disease Groups
Anxiety Disorders
Depressive Disorders
Diabetes Mellitus
Drug Poisoning / Overdose
Heart Failure
Hyperthyroidism
Hypothyroidism
Kidney Disease
Malignant Neoplasms
0.96 (0.94, 0.98) ***
1.04 (0.94, 1.15)
1.04 (0.92, 1.18)
0.92 (0.84, 1.01) .
0.91 (0.78, 1.06)
1 (0.86, 1.16)
1.27 (0.91, 1.78)
0.93 (0.77, 1.13)
0.91 (0.82, 1.02)
0.98 (0.82, 1.16)
0.92 (0.88, 0.97) **
1 (0.78, 1.29)
1.06 (0.77, 1.45)
0.91 (0.73, 1.13)
0.64 (0.43, 0.93) *
0.69 (0.44, 1.07) .
0.73 (0.28, 1.93)
0.77 (0.54, 1.11)
0.7 (0.5, 0.97) *
1.15 (0.75, 1.75)
0.98 (0.96, 1.01)
0.95 (0.85, 1.07)
0.99 (0.86, 1.15)
1.01 (0.87, 1.18)
0.92 (0.74, 1.16)
0.89 (0.7, 1.13)
0.93 (0.54, 1.59)
0.92 (0.76, 1.11)
0.87 (0.72, 1.05)
1.02 (0.84, 1.23)
0.0 0.5 1.0 1.5 2.0
Rate Ratio
(E)
Ethnicity
Hispanic
Multiple/Other/Unknown
Non-Hispanic
All Study Disease Groups
Anxiety Disorders
Depressive Disorders
Diabetes Mellitus
Drug Poisoning / Overdose
Heart Failure
Hyperthyroidism
Hypothyroidism
Kidney Disease
Malignant Neoplasms
1.08 (0.98, 1.18)
1.27 (0.8, 2.02)
0.95 (0.58, 1.57)
1.18 (0.8, 1.72)
0.94 (0.53, 1.65)
2.03 (0.77, 5.36)
1.02 (0.43, 2.42)
0.92 (0.51, 1.66)
1.44 (0.75, 2.77)
1.34 (0.69, 2.6)
0.93 (0.86, 1) *
0.91 (0.63, 1.32)
1.05 (0.68, 1.63)
0.78 (0.57, 1.05)
0.45 (0.28, 0.74) **
0.86 (0.51, 1.44)
1.12 (0.3, 4.17)
1.1 (0.65, 1.86)
0.68 (0.44, 1.04) .
1.12 (0.59, 2.16)
0.97 (0.95, 0.98) ***
1 (0.93, 1.08)
1.03 (0.94, 1.14)
0.94 (0.87, 1.02)
0.91 (0.8, 1.03)
0.94 (0.83, 1.06)
1.16 (0.87, 1.55)
0.91 (0.8, 1.04)
0.89 (0.81, 0.97) *
1 (0.88, 1.13)
0 2 4 6
Rate Ratio

(F)

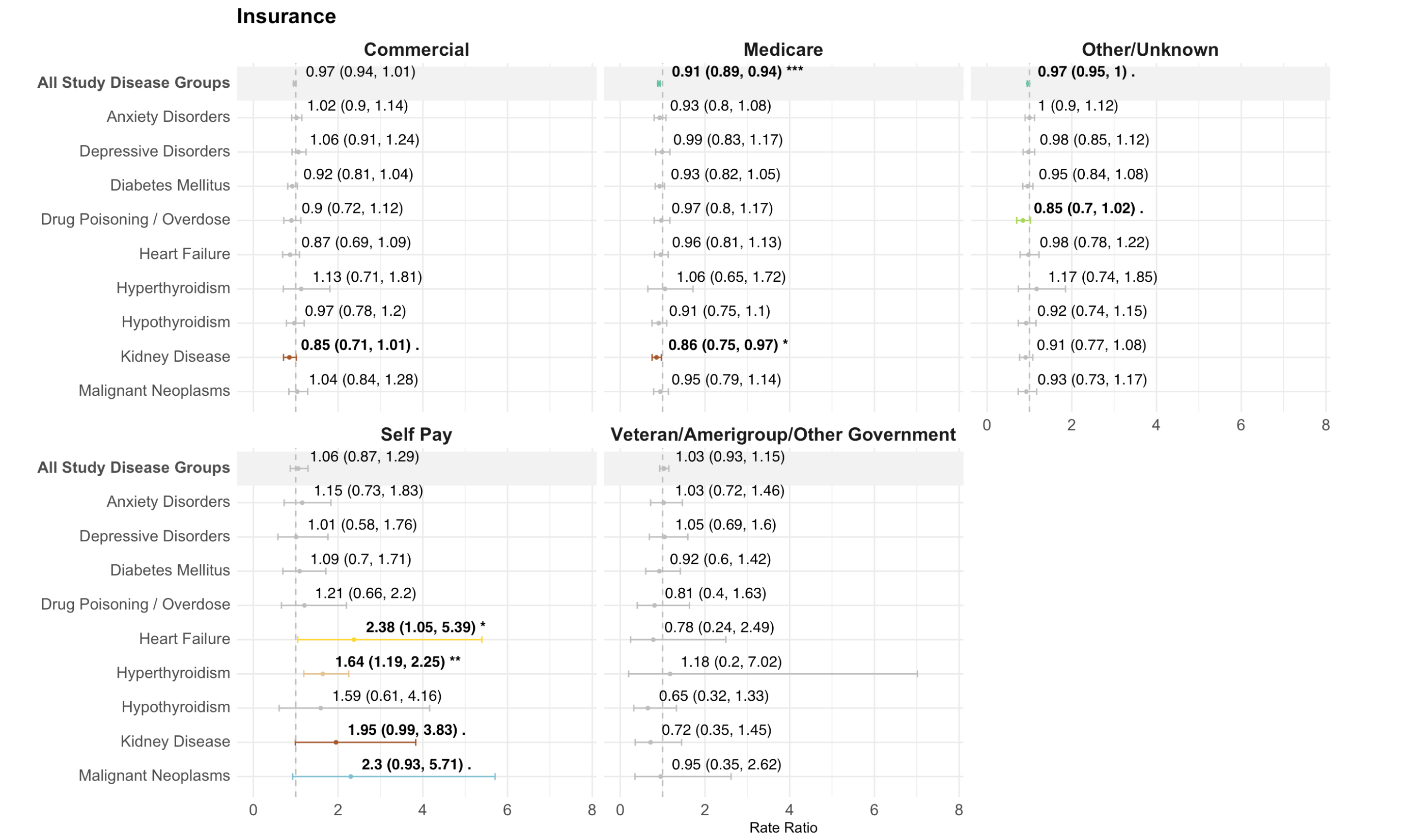
Insurance
Commercial
Medicare
Other/Unknown
Self Pay
Veteran/Amerigroup/Other Government
All Study Disease Groups
Anxiety Disorders
Depressive Disorders
Diabetes Mellitus
Drug Poisoning / Overdose
Heart Failure
Hyperthyroidism
Hypothyroidism
Kidney Disease
Malignant Neoplasms
0.97 (0.94, 1.01)
1.02 (0.9, 1.14)
1.06 (0.91, 1.24)
0.92 (0.81, 1.04)
0.9 (0.72, 1.12)
0.87 (0.69, 1.09)
1.13 (0.71, 1.81)
0.97 (0.78, 1.2)
0.85 (0.71, 1.01) .
1.04 (0.84, 1.28)
0.91 (0.89, 0.94) ***
0.93 (0.8, 1.08)
0.99 (0.83, 1.17)
0.93 (0.82, 1.05)
0.97 (0.8, 1.17)
0.96 (0.81, 1.13)
1.06 (0.65, 1.72)
0.91 (0.75, 1.1)
0.86 (0.75, 0.97) *
0.95 (0.79, 1.14)
0.97 (0.95, 1) .
1 (0.9, 1.12)
0.98 (0.85, 1.12)
0.95 (0.84, 1.08)
0.85 (0.7, 1.02) .
0.98 (0.78, 1.22)
1.17 (0.74, 1.85)
0.92 (0.74, 1.15)
0.91 (0.77, 1.08)
0.93 (0.73, 1.17)
1.06 (0.87, 1.29)
1.15 (0.73, 1.83)
1.01 (0.58, 1.76)
1.09 (0.7, 1.71)
1.21 (0.66, 2.2)
2.38 (1.05, 5.39) *
1.64 (1.19, 2.25) **
1.59 (0.61, 4.16)
1.95 (0.99, 3.83) .
2.3 (0.93, 5.71) .
1.03 (0.93, 1.15)
1.03 (0.72, 1.46)
1.05 (0.69, 1.6)
0.92 (0.6, 1.42)
0.81 (0.4, 1.63)
0.78 (0.24, 2.49)
1.18 (0.2, 7.02)
0.65 (0.32, 1.33)
0.72 (0.35, 1.45)
0.95 (0.35, 2.62)
0
2
4
6
8
Rate Ratio

(G)

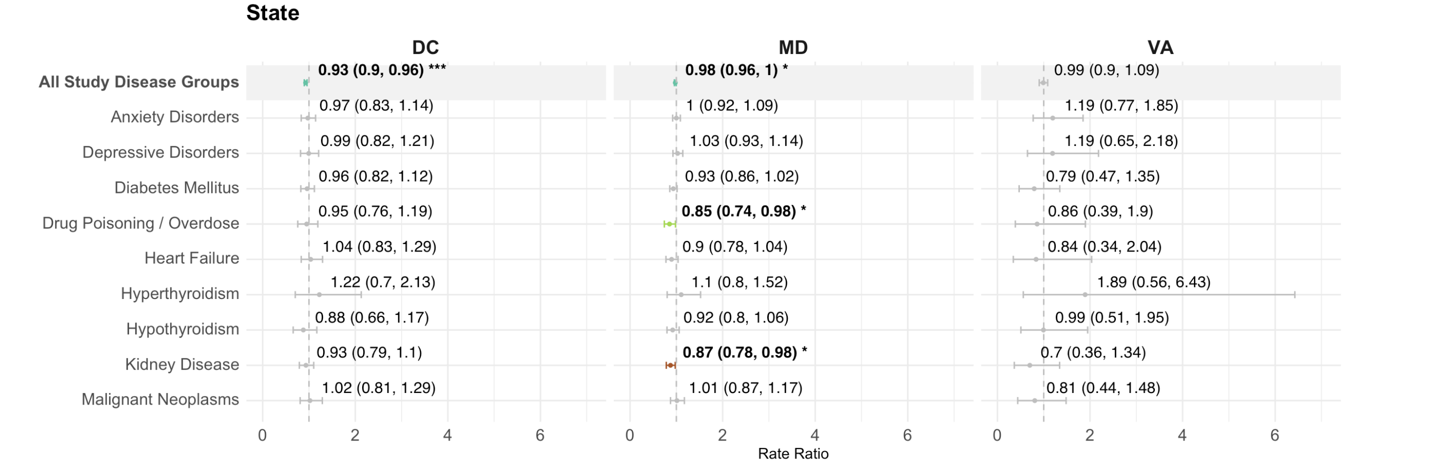
State
DC
MD
VA
All Study Disease Groups
Anxiety Disorders
Depressive Disorders
Diabetes Mellitus
Drug Poisoning / Overdose
Heart Failure
Hyperthyroidism
Hypothyroidism
Kidney Disease
Malignant Neoplasms
0.93 (0.9, 0.96) ***
0.97 (0.83, 1.14)
0.99 (0.82, 1.21)
0.96 (0.82, 1.12)
0.95 (0.76, 1.19)
1.04 (0.83, 1.29)
1.22 (0.7, 2.13)
0.88 (0.66, 1.17)
0.93 (0.79, 1.1)
1.02 (0.81, 1.29)
0.98 (0.96, 1) *
1 (0.92, 1.09)
1.03 (0.93, 1.14)
0.93 (0.86, 1.02)
0.85 (0.74, 0.98) *
0.9 (0.78, 1.04)
1.1 (0.8, 1.52)
0.92 (0.8, 1.06)
0.87 (0.78, 0.98) *
1.01 (0.87, 1.17)
0.99 (0.9, 1.09)
1.19 (0.77, 1.85)
1.19 (0.65, 2.18)
0.79 (0.47, 1.35)
0.86 (0.39, 1.9)
0.84 (0.34, 2.04)
1.89 (0.56, 6.43)
0.99 (0.51, 1.95)
0.7 (0.36, 1.34)
0.81 (0.44, 1.48)
0
2
4
6
Rate Ratio

(H)

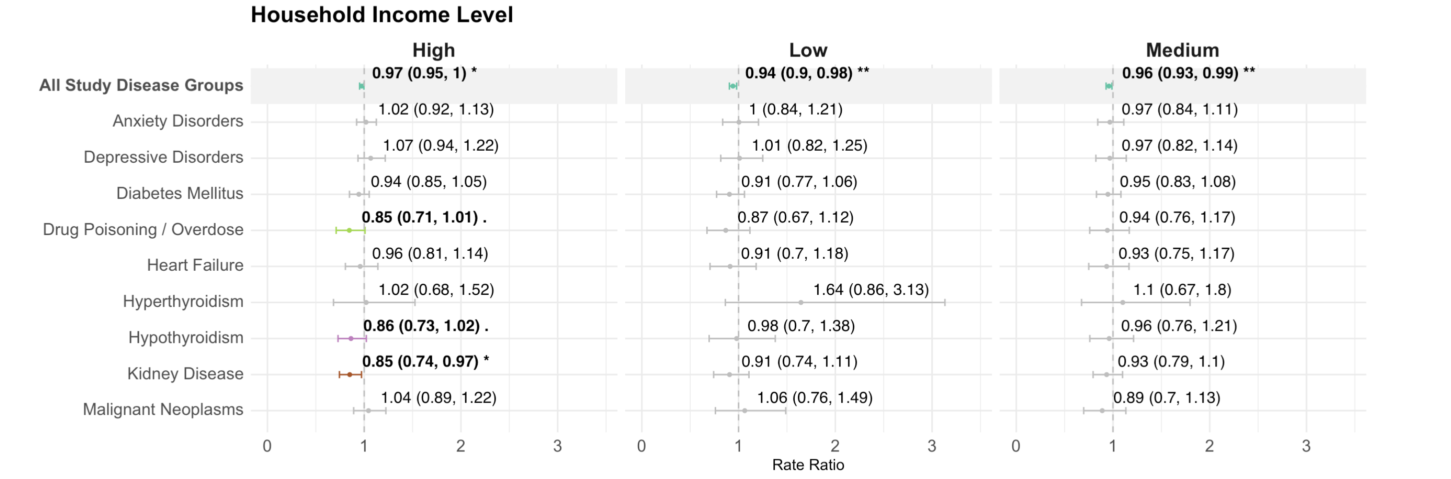
Household Income Level
High
Low
Medium
All Study Disease Groups
Anxiety Disorders
Depressive Disorders
Diabetes Mellitus
Drug Poisoning / Overdose
Heart Failure
Hyperthyroidism
Hypothyroidism
Kidney Disease
Malignant Neoplasms
0.97 (0.95, 1) *
1.02 (0.92, 1.13)
1.07 (0.94, 1.22)
0.94 (0.85, 1.05)
0.85 (0.71, 1.01) .
0.96 (0.81, 1.14)
1.02 (0.68, 1.52)
0.86 (0.73, 1.02) .
0.85 (0.74, 0.97) *
1.04 (0.89, 1.22)
0.94 (0.9, 0.98) **
1 (0.84, 1.21)
1.01 (0.82, 1.25)
0.91 (0.77, 1.06)
0.87 (0.67, 1.12)
0.91 (0.7, 1.18)
1.64 (0.86, 3.13)
0.98 (0.7, 1.38)
0.91 (0.74, 1.11)
1.06 (0.76, 1.49)
0.96 (0.93, 0.99) **
0.97 (0.84, 1.11)
0.97 (0.82, 1.14)
0.95 (0.83, 1.08)
0.94 (0.76, 1.17)
0.93 (0.75, 1.17)
1.1 (0.67, 1.8)
0.96 (0.76, 1.21)
0.93 (0.79, 1.1)
0.89 (0.7, 1.13)
0
1
2
3
Rate Ratio

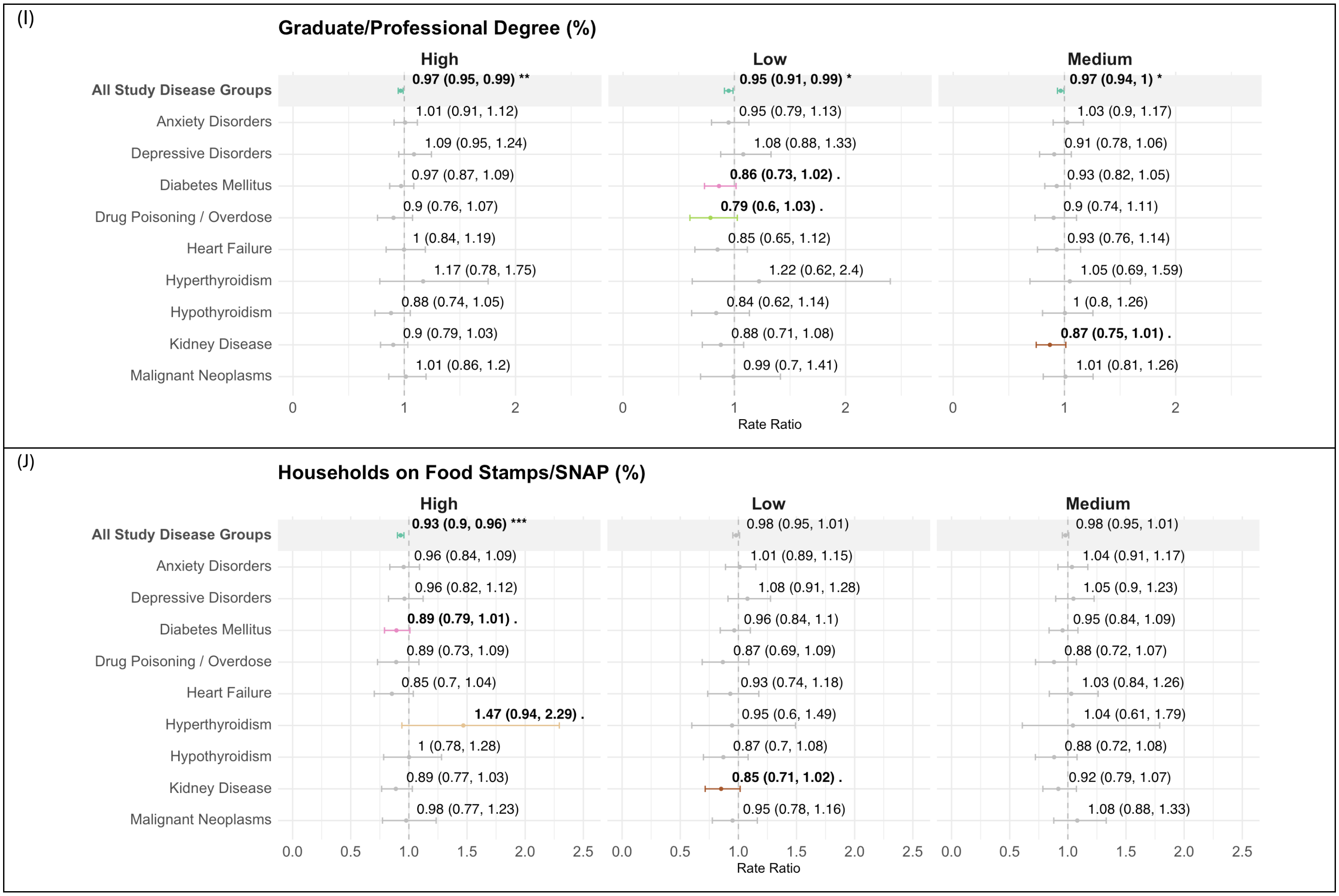
(I)
Graduate/Professional Degree (%)
High
Low
Medium
All Study Disease Groups
Anxiety Disorders
Depressive Disorders
Diabetes Mellitus
Drug Poisoning / Overdose
Heart Failure
Hyperthyroidism
Hypothyroidism
Kidney Disease
Malignant Neoplasms
0.97 (0.95, 0.99) **
1.01 (0.91, 1.12)
1.09 (0.95, 1.24)
0.97 (0.87, 1.09)
0.9 (0.76, 1.07)
1 (0.84, 1.19)
1.17 (0.78, 1.75)
0.88 (0.74, 1.05)
0.9 (0.79, 1.03)
1.01 (0.86, 1.2)
0.95 (0.91, 0.99) *
0.95 (0.79, 1.13)
1.08 (0.88, 1.33)
0.86 (0.73, 1.02) .
0.79 (0.6, 1.03) .
0.85 (0.65, 1.12)
1.22 (0.62, 2.4)
0.84 (0.62, 1.14)
0.88 (0.71, 1.08)
0.99 (0.7, 1.41)
0.97 (0.94, 1) *
1.03 (0.9, 1.17)
0.91 (0.78, 1.06)
0.93 (0.82, 1.05)
0.9 (0.74, 1.11)
0.93 (0.76, 1.14)
1.05 (0.69, 1.59)
1 (0.8, 1.26)
0.87 (0.75, 1.01) .
1.01 (0.81, 1.26)
0
1
2
Rate Ratio
(J)
Households on Food Stamps/SNAP (%)
High
Low
Medium
All Study Disease Groups
Anxiety Disorders
Depressive Disorders
Diabetes Mellitus
Drug Poisoning / Overdose
Heart Failure
Hyperthyroidism
Hypothyroidism
Kidney Disease
Malignant Neoplasms
0.93 (0.9, 0.96) ***
0.96 (0.84, 1.09)
0.96 (0.82, 1.12)
0.89 (0.79, 1.01) .
0.89 (0.73, 1.09)
0.85 (0.7, 1.04)
1.47 (0.94, 2.29) .
1 (0.78, 1.28)
0.89 (0.77, 1.03)
0.98 (0.77, 1.23)
0.98 (0.95, 1.01)
1.01 (0.89, 1.15)
1.08 (0.91, 1.28)
0.96 (0.84, 1.1)
0.87 (0.69, 1.09)
0.93 (0.74, 1.18)
0.95 (0.6, 1.49)
0.87 (0.7, 1.08)
0.85 (0.71, 1.02) .
0.95 (0.78, 1.16)
0.98 (0.95, 1.01)
1.04 (0.91, 1.17)
1.05 (0.9, 1.23)
0.95 (0.84, 1.09)
0.88 (0.72, 1.07)
1.03 (0.84, 1.26)
1.04 (0.61, 1.79)
0.88 (0.72, 1.08)
0.92 (0.79, 1.07)
1.08 (0.88, 1.33)
0.0
0.5
1.0
1.5
2.0
2.5
Rate Ratio

Supplementary Figure 5. Healthcare utilization associated with positive versus negative COVID-19 test results. The difference-in-differences parameter represents the adjusted change in encounter rates comparing post- versus pre-periods, estimated using Poisson regression models overall (A) and by study disease group (B). Bold for p ≤ 0.10. Significance codes: "***" p ≤ 0.001; "**" p ≤ 0.01; "*" p ≤ 0.05; "." p ≤ 0.10.

(A)

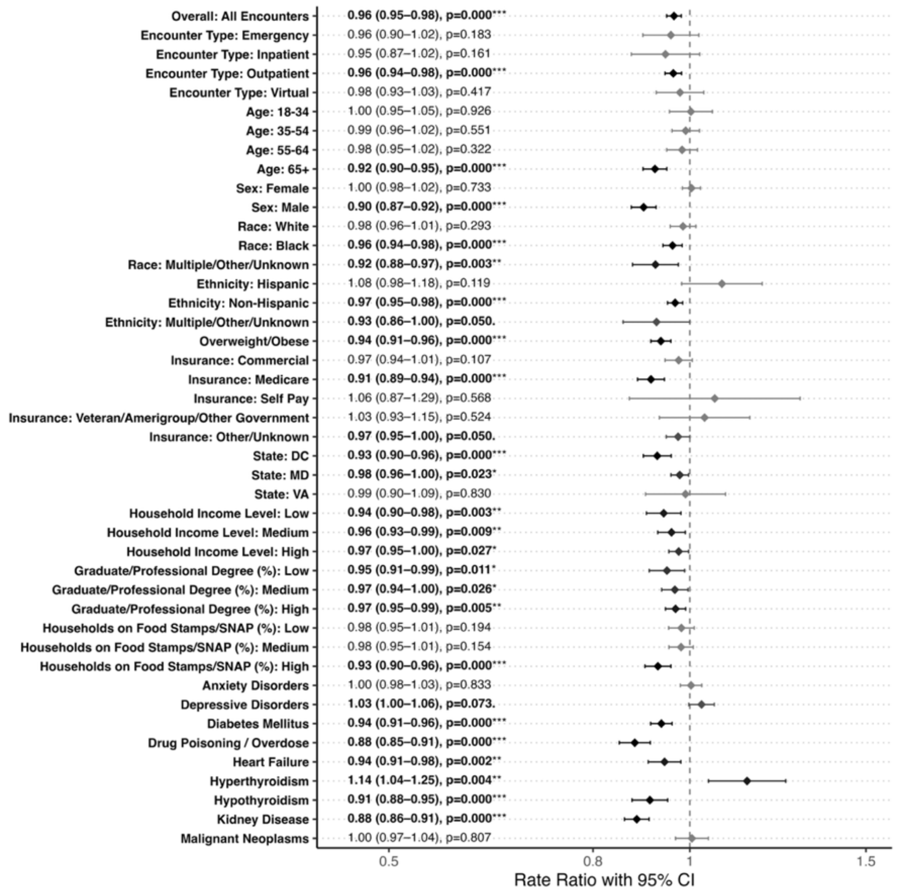

(B)

	Anxiety Disorders	Depressive Disorders	Diabetes Mellitus	Drug Poisoning / Overdose	Heart Failure
Overall: All encounters	1.00 (0.98–1.03), p=0.833	**1.03 (1.00–1.06), p=0.073.**	**0.94 (0.91–0.96), p=0.000*** **	**0.88 (0.85–0.91), p=0.000*** **	**0.94 (0.91–0.98), p=0.002** **
Encounter Type: Emergency	0.91 (0.76–1.08), p=0.280	0.95 (0.75–1.19), p=0.638	0.97 (0.82–1.15), p=0.737	0.83 (0.66–1.06), p=0.132	0.97 (0.71–1.32), p=0.849
Encounter Type: Inpatient	0.93 (0.83–1.06), p=0.282	0.93 (0.81–1.07), p=0.311	0.95 (0.86–1.06), p=0.365	0.90 (0.79–1.03), p=0.119	0.95 (0.83–1.08), p=0.417
Encounter Type: Outpatient	1.01 (0.94–1.09), p=0.740	1.01 (0.92–1.11), p=0.768	0.94 (0.87–1.01), p=0.102	**0.88 (0.78–1.00), p=0.058.**	0.94 (0.83–1.07), p=0.376
Encounter Type: Virtual	0.99 (0.88–1.12), p=0.923	0.95 (0.81–1.11), p=0.515	0.94 (0.83–1.07), p=0.344	1.00 (0.81–1.22), p=0.971	0.99 (0.78–1.26), p=0.966
Age: 18-34	1.03 (0.88–1.21), p=0.717	1.12 (0.90–1.41), p=0.307	1.05 (0.72–1.53), p=0.810	0.71 (0.45–1.12), p=0.143	**0.50 (0.22–1.12), p=0.090.**
Age: 35-54	1.00 (0.88–1.14), p=0.987	1.05 (0.89–1.24), p=0.570	0.96 (0.83–1.11), p=0.583	0.90 (0.71–1.14), p=0.380	1.18 (0.86–1.62), p=0.299
Age: 55-64	1.03 (0.87–1.22), p=0.713	1.00 (0.83–1.22), p=0.971	0.98 (0.85–1.14), p=0.793	0.95 (0.74–1.22), p=0.672	0.89 (0.68–1.16), p=0.397
Age: 65+	0.97 (0.85–1.12), p=0.688	0.97 (0.83–1.15), p=0.752	**0.89 (0.80–0.99), p=0.039***	0.86 (0.72–1.03), p=0.105	0.92 (0.79–1.07), p=0.287
Sex: Female	1.04 (0.96–1.13), p=0.351	1.05 (0.94–1.16), p=0.403	0.97 (0.88–1.06), p=0.509	0.94 (0.80–1.10), p=0.430	0.96 (0.82–1.13), p=0.635
Sex: Male	0.90 (0.78–1.05), p=0.188	0.98 (0.81–1.18), p=0.809	**0.90 (0.80–1.01), p=0.078.**	**0.81 (0.67–0.98), p=0.029***	0.93 (0.78–1.12), p=0.451
Race: White	0.95 (0.85–1.07), p=0.408	0.99 (0.86–1.15), p=0.928	1.01 (0.87–1.18), p=0.900	0.92 (0.74–1.16), p=0.490	0.89 (0.70–1.13), p=0.327
Race: Black	1.04 (0.94–1.15), p=0.482	1.04 (0.92–1.18), p=0.545	**0.92 (0.84–1.01), p=0.083.**	0.91 (0.78–1.06), p=0.212	1.00 (0.86–1.16), p=0.959
Race: Multiple/Other/Unknown	1.00 (0.78–1.29), p=0.971	1.06 (0.77–1.45), p=0.736	0.91 (0.73–1.13), p=0.377	**0.64 (0.43–0.93), p=0.020***	**0.69 (0.44–1.07), p=0.094.**
Ethnicity: Hispanic	1.27 (0.80–2.02), p=0.303	0.95 (0.58–1.57), p=0.855	1.18 (0.80–1.72), p=0.400	0.94 (0.53–1.65), p=0.819	2.03 (0.77–5.36), p=0.153
Ethnicity: Non-Hispanic	1.00 (0.93–1.08), p=0.948	1.03 (0.94–1.14), p=0.504	0.94 (0.87–1.02), p=0.129	0.91 (0.80–1.03), p=0.153	0.94 (0.83–1.06), p=0.297
Ethnicity: Multiple/Other/Unknown	0.91 (0.63–1.32), p=0.631	1.05 (0.68–1.63), p=0.815	0.78 (0.57–1.05), p=0.101	**0.45 (0.28–0.74), p=0.002** **	0.86 (0.51–1.44), p=0.562
Overweight/Obese	0.96 (0.87–1.06), p=0.450	1.01 (0.90–1.14), p=0.835	**0.91 (0.83–0.99), p=0.036***	**0.83 (0.70–0.98), p=0.030***	0.89 (0.76–1.05), p=0.177
Insurance: Commercial	1.02 (0.90–1.14), p=0.778	1.06 (0.91–1.24), p=0.431	0.92 (0.81–1.04), p=0.175	0.90 (0.72–1.12), p=0.338	0.87 (0.69–1.09), p=0.218
Insurance: Medicare	0.93 (0.80–1.08), p=0.338	0.99 (0.83–1.17), p=0.900	0.93 (0.82–1.05), p=0.219	0.97 (0.80–1.17), p=0.748	0.96 (0.81–1.13), p=0.607
Insurance: Self Pay	1.15 (0.73–1.83), p=0.545	1.01 (0.58–1.76), p=0.973	1.09 (0.70–1.71), p=0.704	1.21 (0.66–2.20), p=0.542	**2.38 (1.05–5.39), p=0.039***
Insurance: Veteran/Amerigroup/Other Government	1.03 (0.72–1.46), p=0.890	1.05 (0.69–1.60), p=0.830	0.92 (0.60–1.42), p=0.716	0.81 (0.40–1.63), p=0.555	0.78 (0.24–2.49), p=0.676
Insurance: Other/Unknown	1.00 (0.90–1.12), p=0.977	0.98 (0.85–1.12), p=0.747	0.95 (0.84–1.08), p=0.469	**0.85 (0.70–1.02), p=0.085.**	0.98 (0.78–1.22), p=0.838
State: DC	0.97 (0.83–1.14), p=0.754	0.99 (0.82–1.21), p=0.952	0.96 (0.82–1.12), p=0.593	0.95 (0.76–1.19), p=0.658	1.04 (0.83–1.29), p=0.742
State: MD	1.00 (0.92–1.09), p=0.997	1.03 (0.93–1.14), p=0.611	0.93 (0.86–1.02), p=0.113	**0.85 (0.74–0.98), p=0.026***	0.90 (0.78–1.04), p=0.141
State: VA	1.19 (0.77–1.85), p=0.425	1.19 (0.65–2.18), p=0.573	0.79 (0.47–1.35), p=0.393	0.86 (0.39–1.90), p=0.704	0.84 (0.34–2.04), p=0.692
Household Income Level: Low	1.00 (0.84–1.21), p=0.968	1.01 (0.82–1.25), p=0.929	0.91 (0.77–1.06), p=0.219	0.87 (0.67–1.12), p=0.270	0.91 (0.70–1.18), p=0.487
Household Income Level: Medium	0.97 (0.84–1.11), p=0.644	0.97 (0.82–1.14), p=0.689	0.95 (0.83–1.08), p=0.426	0.94 (0.76–1.17), p=0.587	0.93 (0.75–1.17), p=0.549
Household Income Level: High	1.02 (0.92–1.13), p=0.719	1.07 (0.94–1.22), p=0.331	0.94 (0.85–1.05), p=0.298	**0.85 (0.71–1.01), p=0.062.**	0.96 (0.81–1.14), p=0.636
Graduate/Professional Degree (%): Low	0.95 (0.79–1.13), p=0.551	1.08 (0.88–1.33), p=0.468	**0.86 (0.73–1.02), p=0.076.**	**0.79 (0.60–1.03), p=0.077.**	0.85 (0.65–1.12), p=0.241
Graduate/Professional Degree (%): Medium	1.03 (0.90–1.17), p=0.709	0.91 (0.78–1.06), p=0.226	0.93 (0.82–1.05), p=0.250	0.90 (0.74–1.11), p=0.329	0.93 (0.76–1.14), p=0.500
Graduate/Professional Degree (%): High	1.01 (0.91–1.12), p=0.889	1.09 (0.95–1.24), p=0.223	0.97 (0.87–1.09), p=0.607	0.90 (0.76–1.07), p=0.250	1.00 (0.84–1.19), p=0.978
Households on Food Stamps/SNAP (%): Low	1.01 (0.89–1.15), p=0.862	1.08 (0.91–1.28), p=0.383	0.96 (0.84–1.10), p=0.600	0.87 (0.69–1.09), p=0.221	0.93 (0.74–1.18), p=0.547
Households on Food Stamps/SNAP (%): Medium	1.04 (0.91–1.17), p=0.584	1.05 (0.90–1.23), p=0.559	0.95 (0.84–1.09), p=0.483	0.88 (0.72–1.07), p=0.211	1.03 (0.84–1.26), p=0.780
Households on Food Stamps/SNAP (%): High	0.96 (0.84–1.09), p=0.499	0.96 (0.82–1.12), p=0.624	**0.89 (0.79–1.01), p=0.069.**	0.89 (0.73–1.09), p=0.254	0.85 (0.70–1.04), p=0.112

	Hyperthyroidism	Hypothyroidism	Kidney Disease	Malignant Neoplasms
Overall: All encounters	**1.14 (1.04–1.25), p=0.004** **	**0.91 (0.88–0.95), p=0.000*** **	**0.88 (0.86–0.91), p=0.000*** **	1.00 (0.97–1.04), p=0.807
Encounter Type: Emergency	0.81 (0.52–1.26), p=0.352	0.84 (0.59–1.19), p=0.322	0.90 (0.72–1.14), p=0.379	0.98 (0.65–1.47), p=0.925
Encounter Type: Inpatient	**1.41 (0.94–2.12), p=0.093.**	0.91 (0.73–1.14), p=0.401	0.92 (0.83–1.02), p=0.118	0.98 (0.82–1.18), p=0.854
Encounter Type: Outpatient	1.09 (0.82–1.45), p=0.567	0.92 (0.81–1.05), p=0.210	**0.90 (0.82–1.00), p=0.040***	1.01 (0.89–1.14), p=0.906
Encounter Type: Virtual	1.02 (0.64–1.64), p=0.929	1.08 (0.86–1.35), p=0.492	0.94 (0.80–1.10), p=0.446	1.03 (0.85–1.26), p=0.749
Age: 18-34	0.73 (0.32–1.66), p=0.455	0.80 (0.46–1.39), p=0.429	0.79 (0.46–1.37), p=0.407	1.12 (0.55–2.31), p=0.751
Age: 35-54	1.07 (0.59–1.92), p=0.828	0.87 (0.68–1.11), p=0.268	**0.78 (0.62–0.98), p=0.036***	0.99 (0.73–1.32), p=0.921
Age: 55-64	1.50 (0.79–2.85), p=0.213	1.02 (0.77–1.36), p=0.879	0.92 (0.75–1.12), p=0.417	1.04 (0.80–1.36), p=0.766
Age: 65+	1.13 (0.76–1.68), p=0.539	0.90 (0.75–1.07), p=0.235	0.91 (0.81–1.02), p=0.101	0.99 (0.84–1.16), p=0.866
Sex: Female	1.12 (0.81–1.56), p=0.494	0.91 (0.79–1.05), p=0.215	0.93 (0.82–1.05), p=0.242	1.03 (0.88–1.22), p=0.692
Sex: Male	1.20 (0.72–2.01), p=0.491	0.91 (0.68–1.22), p=0.529	**0.84 (0.74–0.96), p=0.012***	0.96 (0.80–1.16), p=0.684
Race: White	0.93 (0.54–1.59), p=0.794	0.92 (0.76–1.11), p=0.390	0.87 (0.72–1.05), p=0.150	1.02 (0.84–1.23), p=0.865
Race: Black	1.27 (0.91–1.78), p=0.166	0.93 (0.77–1.13), p=0.482	0.91 (0.82–1.02), p=0.113	0.98 (0.82–1.16), p=0.803
Race: Multiple/Other/Unknown	0.73 (0.28–1.93), p=0.529	0.77 (0.54–1.11), p=0.162	**0.70 (0.50–0.97), p=0.034***	1.15 (0.75–1.75), p=0.524
Ethnicity: Hispanic	1.02 (0.43–2.42), p=0.961	0.92 (0.51–1.66), p=0.788	1.44 (0.75–2.77), p=0.275	1.34 (0.69–2.60), p=0.386
Ethnicity: Non-Hispanic	1.16 (0.87–1.55), p=0.312	0.91 (0.80–1.04), p=0.160	**0.89 (0.81–0.97), p=0.012***	1.00 (0.88–1.13), p=0.974
Ethnicity: Multiple/Other/Unknown	1.12 (0.30–4.17), p=0.864	1.10 (0.65–1.86), p=0.714	**0.68 (0.44–1.04), p=0.078.**	1.12 (0.59–2.16), p=0.723
Overweight/Obese	1.10 (0.74–1.63), p=0.646	0.96 (0.81–1.15), p=0.669	**0.84 (0.74–0.95), p=0.005** **	0.90 (0.76–1.08), p=0.256
Insurance: Commercial	1.13 (0.71–1.81), p=0.613	0.97 (0.78–1.20), p=0.767	**0.85 (0.71–1.01), p=0.070.**	1.04 (0.84–1.28), p=0.748
Insurance: Medicare	1.06 (0.65–1.72), p=0.821	0.91 (0.75–1.10), p=0.318	**0.86 (0.75–0.97), p=0.017***	0.95 (0.79–1.14), p=0.557
Insurance: Self Pay	**1.64 (1.19–2.25), p=0.002** **	1.59 (0.61–4.16), p=0.344	**1.95 (0.99–3.83), p=0.053.**	**2.30 (0.93–5.71), p=0.073.**
Insurance: Veteran/Amerigroup/Other Government	1.18 (0.20–7.02), p=0.858	0.65 (0.32–1.33), p=0.237	0.72 (0.35–1.45), p=0.353	0.95 (0.35–2.62), p=0.926
Insurance: Other/Unknown	1.17 (0.74–1.85), p=0.505	0.92 (0.74–1.15), p=0.476	0.91 (0.77–1.08), p=0.275	0.93 (0.73–1.17), p=0.521
State: DC	1.22 (0.70–2.13), p=0.474	0.88 (0.66–1.17), p=0.374	0.93 (0.79–1.10), p=0.422	1.02 (0.81–1.29), p=0.860
State: MD	1.10 (0.80–1.52), p=0.543	0.92 (0.80–1.06), p=0.250	**0.87 (0.78–0.98), p=0.017***	1.01 (0.87–1.17), p=0.863
State: VA	1.89 (0.56–6.43), p=0.305	0.99 (0.51–1.95), p=0.985	0.70 (0.36–1.34), p=0.283	0.81 (0.44–1.48), p=0.489
Household Income Level: Low	1.64 (0.86–3.13), p=0.130	0.98 (0.70–1.38), p=0.905	0.91 (0.74–1.11), p=0.336	1.06 (0.76–1.49), p=0.718
Household Income Level: Medium	1.10 (0.67–1.80), p=0.700	0.96 (0.76–1.21), p=0.735	0.93 (0.79–1.10), p=0.413	0.89 (0.70–1.13), p=0.341
Household Income Level: High	1.02 (0.68–1.52), p=0.922	**0.86 (0.73–1.02), p=0.088.**	**0.85 (0.74–0.97), p=0.018***	1.04 (0.89–1.22), p=0.591
Graduate/Professional Degree (%): Low	1.22 (0.62–2.40), p=0.561	0.84 (0.62–1.14), p=0.252	0.88 (0.71–1.08), p=0.225	0.99 (0.70–1.41), p=0.966
Graduate/Professional Degree (%): Medium	1.05 (0.69–1.59), p=0.825	1.00 (0.80–1.26), p=0.970	**0.87 (0.75–1.01), p=0.072.**	1.01 (0.81–1.26), p=0.938
Graduate/Professional Degree (%): High	1.17 (0.78–1.75), p=0.448	0.88 (0.74–1.05), p=0.166	0.90 (0.79–1.03), p=0.131	1.01 (0.86–1.20), p=0.864
Households on Food Stamps/SNAP (%): Low	0.95 (0.60–1.49), p=0.810	0.87 (0.70–1.08), p=0.215	**0.85 (0.71–1.02), p=0.073.**	0.95 (0.78–1.16), p=0.619
Households on Food Stamps/SNAP (%): Medium	1.04 (0.61–1.79), p=0.877	0.88 (0.72–1.08), p=0.222	0.92 (0.79–1.07), p=0.284	1.08 (0.88–1.33), p=0.461
Households on Food Stamps/SNAP (%): High	**1.47 (0.94–2.29), p=0.092.**	1.00 (0.78–1.28), p=0.991	0.89 (0.77–1.03), p=0.115	0.98 (0.77–1.23), p=0.844

Rate Ratio with 95% CI
